# Cost-effectiveness of one-off upper abdominal CT screening as an add-on to lung cancer screening in England

**DOI:** 10.1038/s41416-025-03043-z

**Published:** 2025-05-14

**Authors:** Chloe Thomas, Laura Heathcote, Yuxiao Sun, Matthew E. J. Callister, Jessica Kitt, Sabrina H. Rossi, Bethany Shinkins, Juliet A. Usher-Smith, Sophie Whyte, Grant D. Stewart

**Affiliations:** 1 Sheffield Centre for Health and Related Research (SCHARR), School of Medicine and Population Health, University of Sheffield, Sheffield, UK; 2 London School of Hygiene and Tropical Medicine, London, UK; 3 Leeds Institute of Health Sciences, University of Leeds, Leeds, UK; 4 Department of Respiratory Medicine, Leeds Teaching Hospitals NHS Trust, Leeds, UK; 5 Department of Surgery, Cambridge Biomedical Campus, University of Cambridge, Cambridge, UK; 6 CRUK Cambridge Centre, Cambridge Biomedical Campus, Cambridge, UK; 7 Division of Health Sciences, Warwick Medical School, University of Warwick, Coventry, UK; 8 Department of Public Health and Primary Care, University of Cambridge, Cambridge, UK; 9 Department of Oncology, Cambridge Biomedical Campus, University of Cambridge, Cambridge, UK; 10 Research and Innovation, Leeds Teaching Hospitals NHS Trust, Leeds, UK; 11 Department of Urology, Leeds Teaching Hospitals NHS Trust, Leeds, UK; 12 Department of Radiology, Leeds Teaching Hospitals NHS Trust, Leeds, UK; 13 Department of Radiology, University of Cambridge, Cambridge, UK; 14 Department of Endocrine Surgery, Leeds Teaching Hospitals NHS Trust, Leeds, UK; 15 Division of Infection, Immunity and Respiratory Medicine, Faculty of Biology, Medicine and Health, The University of Manchester, Manchester, UK; 16 School of AHP & Midwifery, Faculty of Health Studies, University of Bradford, Bradford, UK; 17 Pitcairn Practice, Balmullo Surgery, Fife, UK; 18 Exeter Collaboration for Academic Primary Care (APEx), University of Exeter, Exeter, UK; 19 The Pancreas Unit, Leeds Teaching Hospitals NHS Trust, Leeds, UK; 20 Leeds Vascular Institute, Leeds Teaching Hospitals NHS Trust, Leeds, UK

**Keywords:** Cancer screening, Renal cancer, Kidney diseases, Health care economics

## Abstract

**Background:**

Low-dose computed tomography (CT) screening for lung cancer is available for high-risk individuals in England. Screening simultaneously for upper abdominal conditions, including cancer, is feasible. Here, we estimate the cost-effectiveness of one-off upper abdominal CT screening, added onto lung cancer screening, based on the Yorkshire Kidney Screening Trial (YKST) feasibility study.

**Methods:**

A multi-disease health economic model was developed. Ten cancers and abdominal aortic aneurysm (AAA) were modelled over a lifetime horizon. YKST data informed disease prevalence, resource use and screening costs. Costs, quality-adjusted life-years (QALYs) and cost-effectiveness were estimated probabilistically.

**Results:**

Screening per person costs £70.89, produces 0.0059 QALYs, and has 96% probability of being cost-effective, with an incremental cost-effectiveness ratio of £12,085. AAA contributes most to cost-effectiveness, followed by kidney cancer, but some cancer findings reduce cost-effectiveness. Screening is more cost-effective at younger ages. Screen-detectable disease prevalence, severity and mortality risk contribute most to uncertainty.

**Conclusions:**

One-off upper abdominal CT screening is potentially cost-effective, but costs, harms and benefits vary between conditions. Cost-effectiveness is driven by early diagnosis of AAA, then kidney cancer, illustrating the importance of considering all relevant diseases in screening models. A larger trial would provide more robust data to refine the cost-effectiveness argument.

**Clinical Trial Registration:**

ClinicalTrials.gov: NCT05005195

## Introduction

Targeted screening aimed at high-risk people is an alternative to population-wide screening that is gaining popularity due to its favourable risk-benefit profile. One such programme is the English National Health Service (NHS) lung cancer screening programme, which is currently being rolled out to people with high lung cancer risk as assessed by the Prostate, Lung, Colorectal and Ovarian cancer screening trial risk model or the Liverpool Lung Project risk model, via a biennial low-dose thoracic computer tomography (CT) scan [1]. High lung cancer risk is most commonly associated with smoking history, with ever-smokers also at higher risk for a wide range of other conditions, including other cancers, cardiovascular disease and abdominal aortic aneurysm (AAA) [2], many of which may benefit from early detection. For example, people eligible for lung cancer screening are estimated to have 1.6 fold higher risk of kidney cancer than the general population [3], and ever-smokers are estimated to have a 3.28 fold higher risk of AAA than never-smokers [4]. Attendance for a thoracic CT scan could therefore potentially offer a cost- and time-efficient opportunity to screen for other diseases simultaneously, reducing the need for separate screening programmes.

The Yorkshire Kidney Screening Trial (YKST) aimed to explore the feasibility and potential clinical benefit of offering an upper abdominal non-contrast CT screen [5], as an add-on to a thoracic CT screen within a community-based trial of lung cancer screening; the Yorkshire Lung Screening Trial (YLST) [6]. In YKST, YLST participants who were not diagnosed with cancer in their first screen and were returning for a second scan after two years, were invited to also have an upper abdominal CT scan. Uptake of the abdominal CT scan was high (93%) and 4019 people were scanned [7]. Whilst the study was originally designed to detect kidney cancer, of which it identified ten new cases, most at early stage, it also picked up ten other abdominal cancers of various types, 60 new abdominal aortic aneurisms and over 100 other serious findings [7]. The prevalence of these new findings, which were not identified on the thoracic CT scan, together with high uptake and patient acceptability within this study, suggests that the combined screening approach is feasible and of potential benefit.

Currently there is no English screening programme for kidney cancer, but studies have suggested that potentially large shifts from late to early stage cancer are achievable through screening of asymptomatic patients [8], which would have considerable mortality benefits [9], and reduce NHS spending on costly metastatic kidney cancer treatment [10]. A previous health economic modelling study has suggested that population-wide kidney cancer screening using ultrasound could be cost-effective, but is highly dependent upon cancer prevalence and the stage shift achieved [10]. In contrast, an ultrasound-based screening programme for AAA does already exist in England but is targeted only at men aged 65 [11], as the lower prevalence of AAA in women means it is cost-ineffective in this population [12, 13]. Enriching cancer and AAA prevalence by selecting high-risk patients for targeted upper abdominal screening could therefore potentially improve cost-effectiveness. The aim of this study is to assess whether upper abdominal CT screening as an add-on to lung cancer screening has the potential to be cost-effective from the English NHS perspective, and if so, to inform the design of a full-scale randomised controlled trial to assess potential mortality benefits, by identifying key uncertainties. In line with YKST we model the potential impact of a one-off screen.

## Methods

**Fig. 1:**
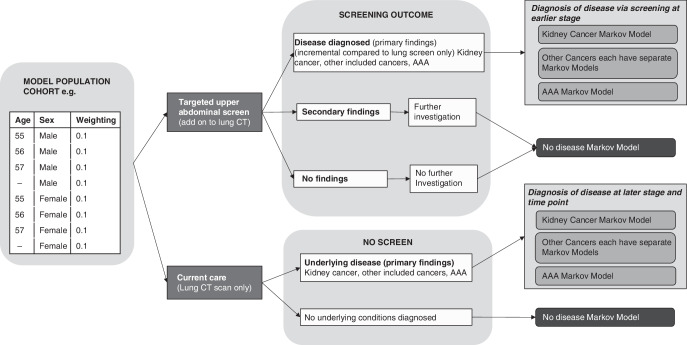
Model structure diagram. Population cohorts are modelled separately and then results are combined through weighting to reassemble the eligible screening population. The decision tree models the short-term process of screening and diagnosis. Lifetime costs and QALYs are gathered through separate Markov models for each included condition, in addition to a ‘no disease’ Markov model.

A new health economic model was developed in R software (version 4.2.1)[14] to evaluate the potential costs and benefits of a one-off upper abdominal screen as an add-on to lung cancer screening. A decision tree structure was used to model the short-term process of screening and diagnosis, with a set of cohort Markov models with annual cycles used to estimate outcomes over a lifetime horizon (Fig. 1). The modelled cohorts represent people who are eligible for and take up upper abdominal screening. People who do not take up screening were not included as they were assumed to incur no costs or benefits incrementally to the lung screen. The population comprises a series of age and sex-specific cohorts, which together cover both the age range eligible for lung cancer screening (55–74) [1], and the age range included in YKST, which was slightly wider (55–81) [5]. Differential weighting of cohorts based on data from YKST enabled a composite population representing likely eligibility in England to be modelled. Full model methods and parameters are available in the supplementary technical methods document.

Although initially designed to screen for kidney cancer, YKST detected a large number of other diseases [7]. It was not feasible to model long-term outcomes for all these findings. Instead, prioritisation of diseases for explicit modelling was based on their screen-detectable prevalence and the likely impact of early diagnosis on healthcare costs, quality of life and survival. Additionally, a small number of rare but serious conditions not found in YKST but expected to be found in a larger study were included (e.g., stomach cancer). The final model included ten cancers defined by organ affected (kidney, liver, stomach, oesophageal, pancreatic, upper urinary tract, colon, adrenal, gallbladder, and lymphomas) and abdominal aortic aneurism (AAA). All other conditions found through YKST (called secondary findings hereon) were modelled only in terms of their short-term diagnostic costs, based on resource data collected through YKST [7]. This included some serious findings such as large kidney stones and Bosniak 2 F, 3 and 4 renal cysts, in addition to minor findings many of which required no follow-up, and false positive findings, which required follow-up but were not found to be serious upon further investigation. Screen-detectable prevalence and predicted stage distribution at screening for cancers by age and sex, was estimated based on published incidence rates [15], current stage distribution [16], and estimates of the time taken for cancer to progress from the start of one stage to the start of the next (dwell time) [17], together with expert input on likely coverage and sensitivity of the CT scan for each organ (see supplementary methods appendix for details). Screen-detectable prevalence and size distribution of AAA by age and sex was informed directly through YKST data, as was screen-detectable prevalence of secondary findings [7]. Whilst some YKST patients had more than one finding at screening, no patients were found to have more than one cancer/AAA, so for simplicity, diseases were modelled independently (this is a limitation that could lead to a small amount of double-counting for benefits). Whilst both model arms were assumed to receive lung screening, this was not explicitly modelled and only the incremental impact of adding the upper abdominal scan to the thoracic scan was assessed. This meant that any findings that would be detected on the thoracic scan as well as on the upper abdominal scan were excluded. The model further excluded pre-existing diagnosed disease, pre-existing undiagnosed disease missed by screening and disease developed post-screening.

Costs of the upper abdominal screen were estimated based on resources used in YKST, modified where appropriate to take into account likely economies of scale if screening were to be rolled out in practice (Table 1). Resource was only included if it was: (a) incremental to use of resources for the lung screen; (b) was specifically required for the clinical pathway rather than e.g., as part of study administration; (c) would be expected to be required in any future roll-out of screening in practice. It was assumed based on expert advice that inviting and consenting patients and performing the scan would in practice incur negligible costs in addition to those used for lung cancer screening, so only costs of scan reporting, informing patients of the result and screening review meetings for positive results were included. Whilst screening review meetings were carried out for minor findings (such as small stones or cysts) in YKST, it was assumed that this would not happen if screening were to be rolled out in practice and that such individuals would incur the costs of a negative rather than a positive finding. YKST resource use data was also used to define a set of standard diagnostic pathways for each positive finding (including secondary findings), which were converted into diagnostic costs using standard cost sources [18]. A small screening harm was also included, applied as a one-off utility decrement of 0.00078 per person, based on a small increased risk of cancer due to the radiation produced by the scan [19, 20] (see supplementary methods for details). No additional decrements were applied for psychological harms as previous research with YKST study participants indicated that there was negligible harm from the addition of the abdominal scan to the thoracic scan [21].

**Table 1:** Resources and costs included for upper abdominal screening as an add-on to lung cancer screening.

Resource	Unit	Unit cost	Cost negative screen	Cost positive screen
Patient invitation and information leaflet	1 invite	£0^a^	£0	£0
Scan time	1 scan	£0^a^	£0	£0
Radiologist reading & reporting of scan	1 scan	£15	£15	£15
Screening review meeting	1 h/15 scans	£217.69	£0	£14.51
- Consultant	1 h	£145		
- Clinical Nurse Specialist Band 6	1 h	£59		
- Admin Staff Band 3	1 h	£13.69		
Patient letter	1 letter	£6.85	£6.85	£6.85
- Clinical Nurse Specialist Band 6	5 min	£4.92		
- Admin Staff Band 3	5 min	£1.14		
- Paper & printing	1 letter	£0.04		
- Stamp 2^nd^ class	1 letter	£0.75		
Patient phone call & referral	1 patient	£3.03	£0	£3.03
- Clinical Nurse Specialist Band 6	2.5 min	£2.46		
- Admin Staff Band 3	2.5 min	£0.57		
**TOTAL**	**1 screen**	**£26.77**^**b**^	**£21.85**	**£39.39**

Staff time costs estimated from [33], all other costs and staff resource use come directly from YKST.

^a^Cost assumed to be absorbed within the existing thoracic scan costs.

^b^Based on weighted average cost positive screen and cost negative screen expected in the population aged 55–74.

Separate Markov models were developed for each of the modelled diseases, together with a ‘no disease’ model with ‘alive’ and ‘dead from all causes’ health states. The latter estimated long-term outcomes for the proportion of the population with secondary findings or no findings at screening, based on age-specific utilities and life expectancy, adjusted for smoking status [22, 23]. All the cancer Markov models were based on a similar structure (see supplementary methods), with health states corresponding to cancer stages I to IV, undiagnosed versus diagnosed disease and death from cancer or other causes, with the baseline population distributed in diagnosed stages in the screening arm, and equivalent undiagnosed stages in the control arm. Transition to later stage diagnosed cancer in the control arm was estimated through calibration to dwell time and current stage distribution data [16, 17], whilst cancer mortality was based on stage-specific survival data [9]. Detailed treatment costs from a previous study for kidney cancer were updated [10]. However, due to a lack of good, comparable cost information for all other cancer types, it was assumed that these applied to all modelled cancers. Utility multipliers were applied by stage and time since diagnosis to reflect reduced quality of life in cancer patients [24]. The AAA Markov model structure and parameters were simplified from a previously published model [12].

An independent researcher not involved directly in model code development checked the code for errors (LH). A set of validations were carried out to ensure that the model replicated the YKST results for disease prevalence, within the expected level of uncertainty (Supplementary Fig. S1; Supplementary Table S1). Model outcomes included costs, life years, quality-adjusted life years (QALYs) and cost-effectiveness, changes in cases and deaths for each of the modelled diseases and estimates of diagnostic resource use. All costs and QALYs were discounted at 3.5% in the basecase analysis in line with NICE guidelines [25].

All model analyses were performed based on 2000 probabilistic runs. Parameters and their distributions are described in the Supplementary Excel file. The basecase analysis assessed the impact of screening in the eligible population and separately by age and sex. It also evaluated the contribution of each modelled disease to the cost-effectiveness results. A maximum justifiable cost analysis was carried out to determine the maximum cost of screening to maintain cost-effectiveness. Scenario analyses were carried out to investigate the impact of key structural uncertainties around cancer stage distribution at screening, cancer mortality with screening diagnosis, cancer prevalence, cancer treatment costs, diagnostic utilities, population composition, disease inclusion and discount rates. Value of information analysis was carried out using the Sheffield Accelerated Value of Information tool to determine the value of reducing parameter uncertainty [26].

## Results

**Fig. 2:**
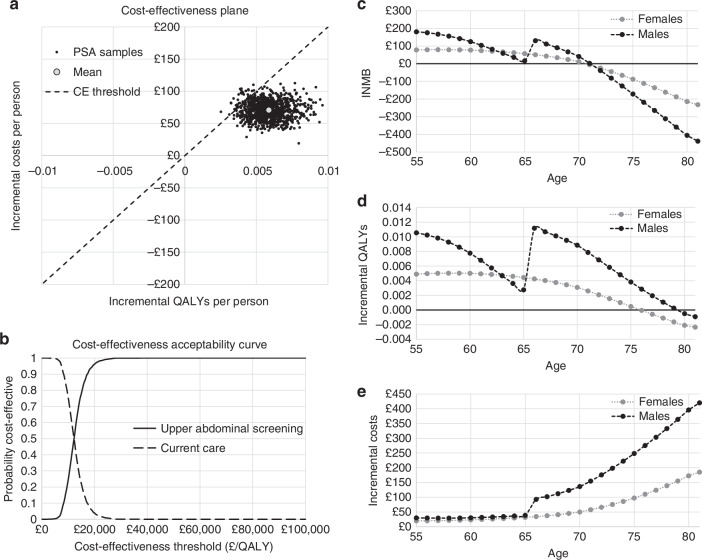
Cost-effectiveness results comparing upper abdominal screening as an add-on to lung screening, against lung screening only (current care) in the basecase analysis. **a** Distribution of probabilistic results (incremental per person costs and QALYs) on the cost-effectiveness plane, for the population aged 55–74. The red point indicates the probabilistic mean and the dotted line represents the £20,000 per QALY threshold; **b** A cost-effectiveness acceptability curve showing the probability that upper abdominal screening is cost-effective at different willingness-to-pay thresholds for the population aged 55–74. Graphs showing how per person incremental cost-effectiveness results change by age and sex of the screening population for **c** incremental net monetary benefit (based on a willingness-to-pay threshold of £20,000 per QALY); **d** incremental QALYs; **e** incremental costs.

Results indicate that, across all included diseases, adding an upper abdominal CT scan to the thoracic CT scan offered within the lung cancer screening programme would be cost-effective with an incremental cost-effectiveness ratio (ICER) of £12,085. This is equivalent to an incremental net monetary benefit (INMB) of £46.42 per person scanned, based on a willingness to pay threshold of £20,000 per QALY (Table 2). At this threshold, the probability screening would be cost-effective is 96%, shown by the distribution of probabilistic results on the cost-effectiveness plane (Fig. 2a, b). Over the lifetime of each individual, abdominal screening would incur total incremental NHS costs of £70.89 per person scanned, 0.0097 additional life years and 0.0059 additional QALYs compared to delivering the thoracic scan alone. Maximum justifiable cost analyses suggest that screening costs could rise to as high as £73.19 (compared with an average of £26.77 currently), and still be cost-effective. Screening prevents 173 deaths from modelled diseases per 100,000 scanned, of which 142 are from AAA, 18 from kidney cancer and 11 from colon cancer (Supplementary Table S2). Screening is also expected to increase total cancer diagnoses by detecting cases in people who would otherwise die of other causes before diagnosis, with e.g., up to 20 additional kidney cancer cases expected per 100,000 people scanned (Supplementary Table S2). However, on average these cases are likely to be at an earlier stage compared to current care diagnoses (Supplementary Fig. S2).

**Table 2:** Cost-effectiveness results comparing upper abdominal screening as an add-on to lung screening (screening arm), to lung screening only (current care arm) in the basecase analysis for the population aged 55–74.

		Current Care	Screening	Incremental
Costs	Mean	£500.02	£570.91	£70.89
	*Lower*	*£384.26*	*£449.44*	*£44.80*
	*Upper*	*£637.21*	*£712.50*	*£98.47*
Life Years	Mean	12.414	12.424	0.0097
	*Lower*	*12.333*	*12.343*	*0.0069*
	*Upper*	*12.494*	*12.503*	*0.0130*
QALYs	Mean	9.349	9.355	0.0059
	*Lower*	*9.173*	*9.178*	*0.0038*
	*Upper*	*9.525*	*9.531*	*0.0084*
Incremental Net Monetary Benefit	Mean			£46.42
	*Lower*			*-£4.95*
	*Upper*			*£101.94*
ICER				£12,085
Probability Cost-Effective				0.962

All results are per person scanned. Lower and upper values represent 95% credible intervals as measured using probabilistic analysis.

*QALY* quality-adjusted life-year, *ICER* incremental cost-effectiveness ratio.

The contribution of each modelled disease or secondary finding to the total INMB is shown in Table 3. AAA makes the biggest contribution to cost-effectiveness, accounting for over half of the positive INMB, with kidney cancer being the second highest contributor. Pancreatic cancer, liver cancer, lymphomas and gallbladder cancer contribute negatively to INMB indicating that finding these conditions through upper abdominal screening is not cost-effective. As expected, all secondary findings contribute negatively to INMB as their detection incurs diagnostic costs but no benefits in the model, as do the population with no disease who incur screening costs and harms with no benefits. The largest negative contribution comes from kidney secondary findings, which is predominantly due to the large number of them found through screening [7]. A breakdown of diagnostic resources required per 100,000 people screened can be found in Supplementary Table S3.

**Table 3:** The contribution of each modelled disease to total INMB per person scanned, in the basecase analysis for the population aged 55–74.

Disease/Organ	Per Person INMB Attributable to AAA or Cancer Findings by Organ	Per Person INMB Attributable to Secondary Findings by Organ	Per Person INMB Attributable to all Findings within Organ
AAA	**£76.02**	£0	**£76.02**
Colon	**£21.43**	*−£0.22*	**£21.21**
Kidney	**£33.75**	*−£30.89*	**£2.86**
Upper urinary tract	**£2.84**	*−£0.12*	**£2.72**
Stomach	**£0.54**	£0	**£0.54**
Oesophagus	£0	£0	£0
Adrenal gland	**£0.48**	*−£0.77*	*−£0.30*
Gallbladder	*−£0.51*	*−£1.15*	*−£1.66*
Lymph nodes & spleen	*−£2.33*	*−£0.06*	*−£2.40*
Liver	*−£6.08*	*−£0.56*	*−£6.64*
Pancreas	*−£18.18*	*−£1.02*	*−19.20*
No disease (screening only)	*−£26.72*		
**TOTAL**	**£46.42**		

Contributions reflect both disease prevalence and impact per person with the disease. Italic text indicates negative contributions to INMB and bold text indicates positive contributions.

*AAA* abdominal aortic aneurysm, *INMB* incremental net monetary benefit assuming a willingness to pay threshold of £20,000/quality-adjusted life-year.

Cost-effectiveness of screening varies significantly by age and sex (Fig. 2c–e and Supplementary Fig. S2). In general, per person costs increase with age, and QALYs reduce with age, with both being slightly higher for men than women. The optimal age for screening men and women in terms of cost-effectiveness would be age 55. It would not be cost-effective to screen people aged over about 70, and screening women aged over 75 and men aged over 79 would lead to QALY loss. Whilst lung screening eligibility is limited to ages 55–74, the YKST study included individuals aged 55–81 [5], and scenario analysis results based on the YKST age range suggest it is not cost-effective to screen this population due to the inclusion of the older age groups (Table 4 and Supplementary Fig. S3). The age curves for men show a sharp discontinuity between age 65 and 66, which is the point at which men become eligible for pre-existing AAA screening in England [11]. There was no evidence in the YKST data for a sharp fall in AAA prevalence after age 65 in men despite the existence of the AAA screening programme [5], which suggests there could be a substantial proportion of this population who either do not take up AAA screening or who develop AAA after age 65. Cost-effectiveness is higher in men aged 66 than those aged 65, as the former have no further opportunity for AAA screening and therefore benefit particularly from the abdominal CT, whilst the latter may have AAA screening within the same year, removing much of the benefit of the abdominal CT.

**Table 4:** Comparison of incremental per person cost-effectiveness results for basecase and scenario analyses.

Scenario Analysis	Incremental Cost	Incremental Life Years	Incremental QALYs	Incremental NMB	ICER	Probability Cost-effective
Basecase	£70.89	0.0097	0.0059	£46.42	£12,085	0.962
1a) Reduced Cancer Stage-Specific Mortality with Screening - as per Cardoso et al. 2022	**−£59.50**	0.0190	0.0124	£307.39	**Dominant**	1.000
1b) Reduced Cancer Stage-Specific Mortality with Screening – half the impact of Cardoso et al. 2022	£16.19	0.0137	0.0087	£157.61	£1863	1.000
2a) Cancer Stage Shifts with Screening Halved	£88.61	0.0079	0.0046	£4.01	£19,134	0.563
2b) % Cancer Stage Four with Screening 25% More	£71.61	0.0096	0.0058	£44.56	£12,329	0.957
2c) % Cancer Stage Four with Screening 25% Less	£70.17	0.0098	0.0059	£48.31	£11,845	0.967
3a) Cancer Metastatic Treatment Costs Halved	£84.25	0.0097	0.0059	£33.07	£14,362	0.922
3b) Metastatic Treatment Costs Halved for all Cancers Apart from Kidney Cancer	£74.45	0.0097	0.0059	£42.86	£12,692	Between 0.922 and 0.962^a^
3c) Metastatic Treatment Costs Doubled	£44.18	0.0097	0.0059	£73.14	£7531	0.987
3 d) Metastatic Treatment Costs Doubled for all Cancers Apart from Kidney Cancer	£63.77	0.0097	0.0059	£53.55	£10,871	Between 0.962 and 0.987^a^
3e) All Cancer Treatment Costs Halved	£77.21	0.0097	0.0059	£40.11	£13,162	0.963
4a) Kidney Cancer Prevalence Doubled	£52.35	0.0110	0.0066	£80.26	£7895	0.996
4b) Kidney Cancer Prevalence Halved	£80.16	0.0090	0.0055	£29.51	£14,619	0.878
4c) All Cancer Prevalence Doubled	£45.65	0.0107	0.0062	£78.55	£7351	0.968
4 d) All Cancer Prevalence Halved	£83.51	0.0092	0.0057	£30.36	£14,667	0.937
5) Utility Decrement Included for Secondary Findings	£70.89	0.0097	0.0043	£15.16	£16,476	0.702
6) YKST Population Age Distribution (55–81)	£106.50	0.0084	0.0048	*−£9.63*	*£21,989*	*0.366*
7a) Discount Rates for Costs and QALYs 1.5%	£61.06	0.0132	0.0084	£106.35	£7295	1.000
7b) Discount Rates for Costs and QALYs 5%	£77.73	0.0077	0.0045	£11.46	£17,430	0.681
8a) No AAA Findings Included in Model	£21.49	0.0010	−*0.0004*	−*£30.05*	*Dominated*	*0.087*
8b) Only AAA Findings Included in Model	£71.08	0.0087	0.0055	£39.35	£12,873	0.999
8c) Only Kidney Findings Included in Model	£25.26	0.0013	*−0.00001*	−*£25.42*	*Dominated*	*0.015*

Incremental results that are cost-saving or dominant compared to current care are highlighted in bold text, whilst incremental results that lose QALYs or life years, or that are not cost-effective compared to current care (assuming a willingness to pay threshold of £20,000 per QALY), are highlighted in italic text.

*QALY* Quality-adjusted life-year, *INMB* Incremental net monetary benefit assuming a willingness to pay threshold of £20,000/QALY, *ICER* Incremental cost-effectiveness ratio, *YKST* Yorkshire Kidney Screening Trial.

^a^Not possible to estimate uncertainty more precisely due to way in which sensitivity analysis results calculated.

Scenario analysis results indicate that the model results are reasonably robust to a range of alternative structural assumptions (Table 4 and Supplementary Fig. S3). Screening would still be cost-effective even if stage shifts are smaller than estimated for the basecase analysis, if cancer treatment costs are significantly lower or higher than expected, or if the discount rate for costs and QALYs is higher. If stage-specific cancer mortality is reduced through screening, then screening could become cost-saving. Cost-effectiveness could also be improved by selecting a subset of people with higher cancer risk for targeted screening, but is reduced if screening identifies fewer cancers than expected or if secondary findings are on average associated with significant reduction in health-related quality of life. Screening would not be cost-effective if the age range for screening is extended up to 81 as per the YKST trial. Scenario analysis around disease inclusion in the model, indicates that if AAA was removed from the model, or if only kidney findings (cancer plus secondary kidney findings) had been modelled, screening would not be cost-effective across the lung screening eligible population (Table 4 and Supplementary Fig. S3), although it would still be cost-effective in the youngest age groups (Supplementary Fig. S4). If only AAA is included in the model then cost-effectiveness is marginally higher than in the basecase analysis (Table 4 and Supplementary Fig. S3).

Expected value of perfect information (EVPI) is £0.38 per person expected to be affected by the decision. The biggest contributors to EVPI are the cancer dwell time parameters (£0.05), which are used to estimate prevalence and stage distribution of cancer at screening, followed by the AAA prevalence and transition probability parameters (£0.03) (Supplementary Table S4). This suggests that results from a larger trial that is sufficiently powered for diseases cases and severity would enable model uncertainty to be reduced.

## Discussion

The findings indicate that adding upper abdominal CT screening to the thoracic CT within the English lung cancer screening programme is potentially cost-effective when looking across multiple conditions (AAA and ten cancers), but the costs, harms and benefits contributed by each modelled condition vary significantly. Probabilistic and structural sensitivity analyses indicate that uncertainty in model results is relatively low and the results are robust to a range of different structural assumptions.

A particular strength of this model is its ability to consider costs and benefits across a large number of different serious screening findings. This novel approach not only increases the validity of the results, but enables investigation into how each disease contributes to total cost-effectiveness, thereby providing important information around how a screening programme might be best developed and targeted to maximise patient benefit and minimise costs. This is particularly important given increasing recognition of potential clinical harms relating to overdiagnosis and overtreatment of incidental findings at diagnostic imaging [27]. For example, whilst screen-detected kidney cancer cases contribute positively to net monetary benefit, the costs associated with the many people with no or only secondary kidney findings overwhelms the benefits in all but men aged under 62. In this case, it is only the inclusion of other cancers and AAA in the model that has enabled screening to be cost-effective across the entire eligible population. Previous cost-effectiveness modelling of focussed renal ultrasound-based screening for kidney cancer similarly found that screening would be cost-effective in 60 year old men, but not in women [10]. Scenario analysis suggests that screening would be more cost-effective if a subpopulation with even higher kidney cancer risk could be identified within the lung screening eligible population (e.g., due to obesity or high blood pressure), or if only a younger subset (e.g., those aged under 65) were screened. However, this would introduce more complexity for implementation and perhaps incur additional costs. Whether or not early detection is beneficial enough to justify a costly screening programme appears to vary widely between cancer types, and our findings for each cancer tend to align with existing studies where these are available. For example; screening for colon cancer is highly cost-effective [28], and in the model contributes significantly to total INMB despite only a small fraction of cases being detectable through upper abdominal screening. Conversely, screening for pancreatic cancer was found not to be cost-effective in the model, and other studies have found that it generally appears to be cost-effective only to screen people with extremely high risk [29].

In the YKST study, upper abdominal screening found three times more new AAA than all cancer cases combined [7], and model results indicate that AAA has an overwhelmingly positive impact on the cost-effectiveness results, such that without it screening would only be cost-effective in people aged under 65. In England, AAA screening is targeted at men aged 65 [11], however despite the high uptake and sensitivity of this programme [12, 30], most of YKST’s new cases were found in men aged over 65, suggesting that there could be scope for expanding AAA screening further. In previous analyses, population screening for AAA was found to be highly cost-effective for men, but marginally not cost-effective for women due partly to their lower AAA prevalence [12, 13]. As smoking increases AAA risk [4], AAA prevalence in the lung cancer screening target population is expected to be higher than in the general population, which may partly explain why the female AAA model results presented here are so cost-effective, together with the cost advantages of adding screening onto the thoracic scan rather than having a separate screening programme. This suggests that while the original purpose of the upper abdominal screening was to detect serious kidney conditions, it could be a mechanism for a relatively cheap and targeted AAA screening in high-risk people, but with additional benefits of cancer detection. If this is the case, then alternate options for expanding AAA screening may need to be evaluated together with the upper abdominal CT screen, to ensure that the optimal screening method is chosen.

Whilst the model’s strength is particularly in its inclusion of multiple serious conditions, it is also limited by the inability to include everything that could have potential long-term costs or benefits from early detection. This is particularly the case for some of the highly prevalent serious kidney findings such as large stones >5 mm and Bosniak cysts grade 2 F or greater, which together contributed to over 100 new findings in YKST [7]. These conditions are not frequently the subject of cost-effectiveness analysis, and it is unclear how much and in which direction, cost-effectiveness results might be altered by their inclusion, although the most serious conditions almost certainly would benefit from early detection. It is also important to note that whilst lung cancer screening will be carried out biennially within the eligible age range in line with cost-effectiveness findings [31], the model developed for this analysis only evaluated the impacts of a one-off screen in line with the YKST study design, and this limits the scope of the model considerably. It is as yet uncertain how an upper abdominal screening programme might be carried out in practice. Repeated screening generally incurs similar screening costs each time, but may produce lower benefit with subsequent screens due to reduced yield or higher benefit with subsequent screens if a lower level of late-stage cancers is found, so it is unclear without further analysis whether repeat screening might be cost-effective. It is also unclear whether positioning of YKST at the second round of lung cancer screening had any impact on the results. The model was also limited by the available data. Good quality, comparable cancer treatment cost data by stage is not available for most of the modelled cancers. Even for kidney cancer where data is of high quality, there is considerable uncertainty around the magnitude of drug costs subject to confidential patient access schemes, although altering costs did not affect model results significantly in scenario analysis. Other data limitations relate to the YKST feasibility trial, which was not powered to reliably inform key parameters around screen-detectable disease cases, severity of disease, or the impact of screening on mortality, and how these vary by age and sex. Instead, these for the most part were calculated within the model based on a range of alternate data sources. The English National Cancer Registration and Analysis Service website is an invaluable resource for a wide range of high quality, open access, consistent data across cancer types [32], which facilitates comparable multi-cancer modelling. However, calculations and assumptions, even if based on excellent data, are no match for statistically significant empirical estimates of screening outcomes. Whilst scenario analysis suggests that model results are fairly robust to altered assumptions around these parameters, a larger upper abdominal screening trial would enable definitive parameter estimates and opportunity to further explore interaction with the existing AAA screening programme.

In conclusion, upper abdominal CT screening as an add-on to lung cancer screening has the potential to be cost-effective when looking across multiple conditions (AAA and ten cancers), but the costs, harms and benefits vary significantly between conditions. Most INMB comes through early detection of AAA followed by kidney cancer, although the impact of finding other types of cancer also contributes, illustrating the importance of considering all relevant diseases in screening models. A larger trial would enable better estimates of screen-detectable disease parameters, improving accuracy of cost-effectiveness estimates.

## Supplementary information

Supplementary Information

Supplementary Methods

Supplementary Results Tables and Figures

Supplementary Parameters file

CHEERS Checklist

## Data Availability

The datasets generated during and/or analysed during the current study are either available in the supplementary information files or available from the corresponding author on reasonable request.

## Code availability

The model code is available under licence for non-commercial purposes upon reasonable request.
