# Supplementary material for: Cost-effectiveness of one-off upper abdominal CT screening as an add-on to lung cancer screening in England
Source: Br J Cancer. 2025 May 14;133(2):239–47. doi: 10.1038/s41416-025-03043-z (PMC12304199; doi:10.1038/s41416-025-03043-z)
Supplement: Supplementary file 1 — Supplementary Information [file 41416_2025_3043_MOESM1_ESM.docx]

**The YKST Study Group full consortium list**

Angela Godoy^1,2^, Fiona Farquhar^3^, Jon Cartledge^4^, Michael Kimuli^4^, Simon Burbidge^5^, Sarah W Burge^1,2^, Iztok Caglic^6^, Emma Collins^7^, Philip AJ Crosbie^8^, Claire Eckert^9^, Sheila Fraser^7^, Neil Hancock^9^, Gareth R Iball^10^, Catriona Marshall^9^, Golnessa Masson^11^, Richard D Neal^12^, Suzanne Rogerson^3^, Andrew Smith^13^, Irene Simmonds^9^, Tom Wallace^14^, Matthew Ward^11^, Matthew EJ Callister^9,15^, Jessica Kitt^16^, Sabrina H Rossi^2,16^, Bethany Shinkins^9,17^, Juliet A Usher-Smith^2,18^, Grant D Stewart^2,16^

^1^Department of Oncology, University of Cambridge, Cambridge Biomedical Campus, Cambridge, CB2 0QQ, UK,

^2^CRUK Cambridge Centre, Cambridge Biomedical Campus, Cambridge, CB2 0QQ, UK,

^3^Research and Innovation, Leeds Teaching Hospitals NHS Trust, Leeds, LS9 7TF, UK

^4^Department of Urology, Leeds Teaching Hospitals NHS Trust, Leeds, UK, Leeds, LS9 7TF, UK

^5^Department of Radiology, Leeds Teaching Hospitals NHS Trust, Leeds, UK, Leeds, LS9 7TF, UK

^6^Department of Radiology, University of Cambridge, Cambridge, CB2 0QQ, UK

^7^Department of Endocrine Surgery, Leeds Teaching Hospitals NHS Trust, Leeds, LS9 7TF, UK

^8^Division of Infection, Immunity and Respiratory Medicine, Faculty of Biology, Medicine and Health, The University of Manchester, Manchester, M13 9NT, UK

^9^Leeds Institute of Health Sciences, University of Leeds, Leeds, LS2 9LN, UK

^10^School of AHP & Midwifery, Faculty of Health Studies, University of Bradford, Bradford, BD7 1DP, UK

^11^Pitcairn Practice, Balmullo Surgery, Fife, KY16 0DZ, UK

^12^Exeter Collaboration for Academic Primary Care (APEx), University of Exeter, Exeter, EX2 4TH, UK

^13^The Pancreas Unit, Leeds Teaching Hospitals NHS Trust, Leeds, LS9 7TF, UK

^14^Leeds Vascular Institute, Leeds Teaching Hospitals NHS Trust, Leeds, UK, Leeds, LS9 7TF, UK

^15^Department of Respiratory Medicine, Leeds Teaching Hospitals NHS Trust, Leeds, LS9 7TF, UK

^16^Department of Surgery, University of Cambridge, Cambridge Biomedical Campus, Cambridge, CB2 0QQ, UK

^17^Division of Health Sciences, Warwick Medical School, University of Warwick, Coventry, CV4 7AL, UK

^18^Department of Public Health and Primary Care, University of Cambridge, Cambridge, CB2 0SR, UK
