# Supplementary material for: Cost-effectiveness of one-off upper abdominal CT screening as an add-on to lung cancer screening in England
Source: Br J Cancer. 2025 May 14;133(2):239–47. doi: 10.1038/s41416-025-03043-z (PMC12304199; doi:10.1038/s41416-025-03043-z)
Supplement: Supplementary file 2 — Supplementary Methods [file 41416_2025_3043_MOESM2_ESM.docx]

**Supplementary Methods**

**Cost-effectiveness of upper abdominal CT screening for kidney cancer as an add-on to targeted lung screening in England**

**Contents**

[1. Model scope 2](#_Toc191628397)

[1.1 Intervention & comparator 2](#_Toc191628398)

[1.1 Eligible population 2](#_Toc191628399)

[1.2 Model outcomes 2](#_Toc191628400)

[1.3 Model perspective 2](#_Toc191628401)

[1.4 Diseases included within the model scope 4](#_Toc191628402)

[2. Model structure 5](#_Toc191628403)

[2.1 Baseline population cohorts 5](#_Toc191628404)

[2.2 Short-term decision tree 5](#_Toc191628405)

[2.3 Long-term Markov models 5](#_Toc191628406)

[2.4 Justification for model structure 6](#_Toc191628407)

[2.5 Model coding, checking and validation 6](#_Toc191628408)

[2.6 Model inputs overview 6](#_Toc191628409)

[3. Decision tree probabilities: Disease prevalence & severity 8](#_Toc191628410)

[3.1 Estimation of screen-detectable cancer prevalence at baseline 8](#_Toc191628411)

[3.2 Estimation of cancer stage distribution at baseline 14](#_Toc191628414)

[3.3 Estimation of screen-detectable prevalence & size of AAA at baseline 15](#_Toc191628415)

[3.4 Estimation of screen-detectable prevalence of incidental findings at baseline 17](#_Toc191628416)

[4. Markov model health states and transitions 18](#_Toc191628417)

[4.1 No disease Markov models 18](#_Toc191628418)

[4.2 Cancer Markov models 18](#_Toc191628420)

[4.3 AAA Markov models 23](#_Toc191628425)

[4.4 All/other cause mortality 26](#_Toc191628428)

[5. Resource use, cost and utility inputs 27](#_Toc191628429)

[5.1 Resource use and costs 27](#_Toc191628430)

[5.2 Utilities 35](#_Toc191628435)

[6. Model analyses 39](#_Toc191628440)

[6.1 Primary model analyses 39](#_Toc191628441)

[6.2 Scenario analyses 39](#_Toc191628442)

[6.3 Value of information analyses 40](#_Toc191628443)

[7. References 41](#_Toc191628444)

# Model scope

## Intervention & comparator

The intervention arm receives upper abdominal screening in addition to a lung screen. The comparator arm receives lung screening only. However, only the incremental impact of adding the upper abdominal screen to the lung screen is assessed in this model. All costs and consequences of upper abdominal screening included in this modelling exercise are assumed to be incurred in addition to the existing lung cancer screening programme. This means that any costs associated with lung cancer screening are excluded, as are any health conditions that would be detected on the lung scan as well as on the upper abdominal scan. A diagram showing the impacts of the modelled intervention on NHS costs and health related quality of life (HRQoL) is shown in Figure 1. This initial study considered a one-off screen only, as evidence was not available to inform the potential benefits of multiple screening rounds.

## Eligible population

The modelled population are those individuals who are eligible for lung cancer screening. The eligible population for lung cancer screening are aged between 55 and 74 and have a history of smoking (either current or past smokers), with a certain level of risk as determined by a number of different risk assessment tools [1-10]. However, YKST included people aged up to 81 in their study, so the modelled age range includes people aged between 55 and 80.

## Model outcomes

Model outcomes include incremental lifetime costs/cost-savings, life years (LYs) and quality–adjusted life years (QALYs), which are used to calculate cost-effectiveness using incremental cost-effectiveness ratios (ICERs) and incremental net monetary benefit (NMB). Other modelled outcomes also include changes in cases and deaths for each of the modelled diseases and estimates of resource use for key resources in the diagnostic pathway.

## Model perspective

The model takes the English National Health Service (NHS) perspective for costs and benefits.

Figure 1: Overview of the modelled impacts of the intervention on NHS costs and HRQoL Impacts included in the model are shown in black text, whilst impacts considered but not modelled are shown in grey text.

## Diseases included within the model scope

YKST found a large number of different previously undetected diseases. Furthermore, there are additional rare but serious conditions that were not found, but that UA screening potentially could find if carried out at scale e.g. adrenal cancer. There is also the potential for some people to be diagnosed with mild conditions that may have no benefit to being detected early, but may require follow-up procedures in order to diagnose properly, which may be costly and potentially could result in harms to health.

It was not feasible to incorporate every single disease into this preliminary model, so a prioritisation exercise was carried out to determine which diseases to include and which to exclude (Figure 2). The following points were considered:

- Whether diagnosis is likely to incur significant additional resource costs or lead to health benefits/harm per individual diagnosed.
- Disease prevalence.
- Whether there is a significant chance of the UA screen finding the condition incremental to the lung screen.

On this basis, the model was designed to incorporate short and long-term costs and benefits for range of different cancers (kidney, liver, stomach, oesophagus, pancreas, colon, ureteric, gallbladder, adrenal & lymphoma), plus abdominal aortic aneurism (AAA). Full modelling of these conditions used data from a variety of different sources, to complement that obtained through YKST. All other conditions were considered to be incidental findings for the purposes of the model and were modelled solely in terms of their short-term resource use and costs, based directly on data gathered by YKST. Future versions of the model may explicitly model a greater number of different incidental findings than was possible for this preliminary version.

Figure 2: Prioritisation exercise for determining which diseases to include in the modelling.

# Model structure

A cohort model was developed with a simple decision tree structure to model the process of screening and diagnosis, followed by a Markov structure to model long-term implications of screening for each disease diagnosed through screening, and for the population with no disease. A high-level diagram of the model structure is shown in Figure 1 of the main manuscript

## Baseline population cohorts

The model population represents all those who undergo upper abdominal screening; i.e. they are eligible for screening (based on the criteria defined above in Model scope), and have chosen to take it up. Some eligible people may be invited to screening but choose not to take it up; however, such people are assumed to incur no screening cost incremental to the lung scan (as the invitation in practice would be to both scans), and so these people are not explicitly modelled.

A set of cohorts are modelled, one for each age and sex within the eligible age group. This enables results to be obtained by age and sex, thereby enabling the model to answer questions around optimal screening age. Alternatively, any eligible population can be represented through weighting of the age/sex results. In the basecase analysis, the eligible population of England was represented by a cohort aged 55-74 with age/sex proportions based on those from YKST. An alternative analysis whereby the full YKST population (age 55-81) was included, enabled modelling of the cost-effectiveness of the trial.

Only disease found through screening is modelled, so it is assumed at baseline that no one has previously diagnosed disease.

## Short-term decision tree

The decision tree models screening and its immediate consequences in diagnosing proportions of the population with each disease.

In the screening arm of the model, decision tree probabilities represent the proportions of the population cohort who get diagnosed through screening with each modelled disease or incidental finding. Each disease is modelled independently and it is assumed that no one is diagnosed with more than one disease. In the screening arm, only disease/incidental findings diagnosed through screening is modelled, so it is assumed that no one has pre-existing diagnosed disease and no one remains with undiagnosed disease following screening. Screening and disease/incidental finding diagnosis are associated with resource use and costs. Screening (but not diagnosis) is assumed to incur a small utility decrement. The remainder of the population cohort have no screen-detectable disease and are assumed to incur no additional resource use, costs or utility decrements beyond those incurred for screening itself.

In the comparator arm, decision tree probabilities for disease from the screening arm, are reused to represent the proportions of the population cohort who have undiagnosed disease. The population with one of the explicitly modelled diseases (cancer or AAA) may be diagnosed later in the long-term Markov model at which point they will incur the relevant resource use, costs and short-term utility decrements. The remainder of the population cohort (including those who would have been diagnosed with incidental findings in the screening arm) are treated as though they have no disease in the long-term model and incur no resource use, costs or utility decrements.

## Long-term Markov models

The Markov model comprises a set of models, one for each true positive disease included in the model, and one for no disease. Incidental finding diagnoses are assumed to have only a short-term impact in the model and therefore they do not require separate long-term Markov models, with outcomes instead being taken from the no disease Markov model. All Markov models have annual cycles and a lifetime horizon.

Each disease Markov model has a bespoke set of health states representing both diagnosed and undiagnosed disease of varying severity levels, in addition to disease specific and other cause mortality health states. In the screening arm, the population is distributed between diagnosed health states at model start, whilst in the comparator arm, the population is distributed between severity levels in the same way, but in the equivalent undiagnosed health states. The no disease Markov model simply has two health states; alive and dead from all causes. In this model, the entire population is in the alive health state at model start.

Transition probabilities include the probability of moving to a more severe disease health state, the probability of being diagnosed (through symptoms or chance) and the probability of dying. Completed Markov models are half-cycle corrected. All living health states accumulate LYs and QALYs, whilst some disease health states may also incur costs of treatment or surveillance. Transitions between health states may also incur costs or utility decrements. Costs, QALYs and LYs are discounted over time and aggregated to produce lifetime estimates for each disease and model arm. Outcomes for each disease are then multiplied by the relevant decision tree probabilities (representing the proportion with each disease or with no disease) so that the relative contribution of each disease to the overall outcome can be assessed.

## Justification for model structure

There are several reasons why a cohort Markov structure was chosen over an individual patient simulation model. The primary reason is that using a cohort model avoids the inclusion of first order uncertainty in the model. Given that the intervention could lead to early detection of a large variety of rare events, a large number of individuals would need to be simulated to smooth out the impacts of early diagnosis. This could lead to very long model run times. This can be avoided using a cohort structure. Secondly, whilst a large number of different downstream conditions are modelled, each condition is rare and so can be considered separately without the need to model feedback loops or interactions between them. This means that it is appropriate to use a standard Markov structure with mutually exclusive health states.

Modelling a cohort does limit model flexibility for modelling population subgroups (e.g. high-risk groups); however, the chosen structure means that differences by age, sex and overall risk (or underlying prevalence) of any of the modelled conditions can still be represented.

## Model coding, checking and validation

The model was built in R software. An independent researcher not involved directly in model build checked the model code and outputs. A set of validations were carried out to ensure that the model replicated the YKST trial results for disease prevalence, within the expected level of uncertainty.

## Model inputs overview

Data informing model inputs and relationship between model parameters is summarised in Figure 3. These are described fully in the following sections.

Figure 3: Data informing model inputs and relationship between model parameters

# Decision tree probabilities: Disease prevalence & severity

Decision tree probabilities represent the proportions of each population cohort who get diagnosed through screening with each modelled disease or incidental finding. The small size of the YKST pilot trial meant high uncertainty around the total amount of disease likely to be found specifically through upper abdominal screening, and it was unable to inform prevalence of rarer conditions, severity of discovered disease, or differences by age and sex. Methods were developed to enable estimation of screen-detectable disease prevalence & severity by age and sex at baseline.

## Estimation of screen-detectable cancer prevalence at baseline

A consistent method was used to estimate screen-detectable prevalence by age and sex across all cancers included in the modelling, for the target population. This first estimated the total undiagnosed prevalence of cancer in the target population, and then combined this with estimates of screening sensitivity to obtain screen-detectable disease estimates.

### Estimating undiagnosed cancer prevalence

Cancer incidence data for England from the National Disease Registration Service (NDRS) was used as the basis for prevalence estimates [11]. NDRS incidence data is available by sex and 5-year age group. Incidence rates per 100,000 were extracted from the 2019 dataset; this year was chosen as more recent data was likely to be affected by the COVID-19 pandemic. Table 1 shows the precise disease definition and ICD-10 codes for each set of incidence data extracted. Incidence rates for each year of age were estimated through linear interpolation of age group data between the mid-points of each age group.

Table 1: Disease definition and ICD-10 codes for cancer incidence data extracted from NDRS 2019 [11].

| **Cancer** | **Definition** | **ICD-10 Code** |
| --- | --- | --- |
| Kidney Cancer | Includes kidney but not renal pelvis | C64 |
| Liver Cancer | Includes liver and intrahepatic bile ducts | C22 |
| Stomach Cancer | Includes stomach | C16 |
| Oesophageal Cancer | Includes oesophagus | C15 |
| Pancreatic Cancer | Includes pancreas | C25 |
| Colon Cancer | Includes colon but not rectum/rectosigmoid junction | C18 |
| Ureteric Cancer | Includes renal pelvis and ureter | C65-66 |
| Gallbladder Cancer | Includes gallbladder | C23 |
| Adrenal Cancer | Includes adrenal gland | C74 |
| Hodgkin Lymphoma | Includes Hodgkin lymphoma | C81 |
| Non-Hodgkin Lymphoma | Includes all non-Hodgkin lymphoma | C82-86 |

The target population includes people at high risk of lung cancer due to a history of smoking. However, smoking is also a risk factor for all of the cancers included in the UA model, meaning that expected incidence amongst the target population is likely to be higher than that expected in the general population. Relative risks (RRs) for cancer in smokers compared to never smokers were identified from a UK review across multiple cancer types and exposures [12]. For each cancer type, RRs were extracted from the original data source together with confidence intervals (see Table 2). For a small number of cancer types, no data was available from the review, so rapid searches were carried out to find sources to inform these. For most cancers, different RRs are given for current and former smokers. A weighted average RR for ever versus never smokers for each cancer was calculated based on the ratio of current to former smokers that participated in YKST (30.5% current smokers). The final step was to estimate the RR in ever smokers (as represented in YKST) compared to the average English population, using the following formula:

RR (ever vs average) = RR (ever vs never) ^ (% never smokers)

The percentage of never smokers was taken from ONS data about adult smoking habits in England from 2021, for men and women aged 16 and over [13]. The calculated RRs for ever smokers versus the average population are found in Table 2. Incidence rate data was then multiplied by these RRs to estimate revised incidence rates for the target population of current/past smokers.

Table 2: Relative risks for cancers in smokers versus various comparators. Data taken directly from published sources is given in black, whilst calculated values are given in grey. Confidence intervals are not given for calculated values as uncertainty comes from more than one source and is combined within the model in probabilistic sensitivity analysis.

| **Cancer Type** | **Sex** | **Current vs Never (95% CI)** | **Former vs Never (95% CI)** | **Ever vs Never (95% CI)** | **Ever vs Average** | **Source** |
| --- | --- | --- | --- | --- | --- | --- |
| Kidney | M | 1.35 (1.13-1.96) | 1.22 (1.09-1.40) | 1.26 | 1.12 | [1] |
|  | F | 1.35 (1.13-1.96) | 1.22 (1.09-1.40) | 1.26 | 1.15 |  |
| Liver | M | 1.61 (1.38-1.89) | 1.47 (1.19-1.82) | 1.51 | 1.23 | [2] |
|  | F | 1.86 (1.33-2.60) | 1.45 (0.80-2.65) | 1.56 | 1.31 |  |
| Stomach | M | 1.62 (1.50-1.75) | 1.34 (1.22-1.47) | 1.42 | 1.19 | [3] |
|  | F | 1.20 (1.01-1.43) | 1.16 (0.92-1.46) | 1.17 | 1.10 |  |
| Oesophageal ** | M | 2.32 (1.96-2.75) | 1.62 (1.40-1.87) | 1.81 | 1.34 | [4] |
|  | F | 2.32 (1.96-2.75) | 1.62 (1.40-1.87) | 1.81 | 1.43 |  |
| Pancreatic | M | 2.20 (1.71-2.83) | 1.17 (1.02-1.34) | 1.19 | 1.19 | [5] |
|  | F | 2.20 (1.71-2.83) | 1.17 (1.02-1.34) | 1.19 | 1.24 |  |
| Colon | M | 1.11 (1.02-1.20) | 1.15 (1.07-1.25) | 1.14 | 1.07 | [6] |
|  | F | 1.11 (1.02-1.20) | 1.15 (1.07-1.25) | 1.14 | 1.08 |  |
| Ureteric*** | M | 3.44 (2.67-4.22) | 1.92 (1.57-2.27) | 2.29 | 1.51 | [7] |
|  | F | 3.56 (2.76-4.36) | 2.04 (1.66-2.42 | 2.42 | 1.71 |  |
| Gallbladder  **** | M | 1.18 (0.98-1.43) | 0.98 (0.84-1.14) | 1.04 | 1.02 | [8] |
|  | F | 1.18 (0.98-1.43) | 0.98 (0.84-1.14) | 1.04 | 1.02 |  |
| Adrenal**** | M | No data | No data | 1.67 (1.45-1.93)* | 1.18 | [9] |
|  | F | No data | No data | 1.67 (1.45-1.93)* | 1.23 |  |
| Hodgkin**** | M | No data | No data | 1.15 (1.02-1.30) | 1.07 | [10] |
|  | F | No data | No data | 1.15 (1.02-1.30) | 1.09 |  |
| Non-Hodgkin**** | M | No data | No data | 1.05 (1.00-1.09) | 1.02 | [10] |
|  | F | No data | No data | 1.05 (1.00-1.09) | 1.03 |  |
| M = male; F = female; CI = confidence interval; *Only values for current vs not current smoker found. These have been assumed to be similar to ever vs never smoker; ** Values for oesophageal adenocarcinoma used as most prevalent UK type. *** Data based on values for urothelial cancers (includes bladder & ureteric cancer). **** Not available from review so found through rapid searching; | | | | | | |

Total prevalence of cancer expected at each age was estimated by calculating cumulative incidence rates (as the population ages) and then ‘offsetting’ these by a number of years equivalent to estimated average cancer sojourn time (defined as the time between when a cancer can be detected to when it is diagnosed) to take into account that each person diagnosed with cancer will have had it for a certain time period before their diagnosis (Figure 4). Note that it is expected that some people have cancer who are not diagnosed before death from other causes, and hence total prevalence might be expected to be greater than these estimates. However, this is already taken into account in the calculations, as the incidence rate denominator is per 100,000 people of the given age (rather than per 100,000 born). The offsetting relies on the assumption that anyone who would have died before diagnosis would have had the same incidence rate as those still surviving until their age of diagnosis, and so this extra prevalence is taken into account. Technically, it should be possible to calculate prevalence by stage using a similar process, which would provide more accurate prevalence (and stage distribution at diagnosis) estimates. This was not implemented here due to lack of time, but should be considered in future model updates.

Figure 4: Method used to calculate cancer prevalence.

Average sojourn time was estimated using two data sources. Firstly; a study that elicited dwell time (the time it takes cancer to progress from the start of one stage to the start of the subsequent stage) for various cancer types, through expert opinion using the Delphi method [14], and secondly; the current stage distribution at diagnosis. The Delphi study estimated median and range of dwell times for stage I, stage II and stage III. For the purposes of the model, the median time was used as the mode in a triangular distribution with the range representing the lower and upper limits. Mean values for each dwell time were estimated by summing the three values given and dividing by three. It was assumed that on average, people would be diagnosed in the middle of each stage, and that stage IV would not last more than a year without being diagnosed (i.e. average time in stage IV before diagnosis is 6 months). Using these assumptions, an estimate of mean sojourn time was calculated for people in each stage for each cancer type (Table 3), with sampling of dwell times for each cancer stage being assumed to be independent in probabilistic analysis. An average estimate of sojourn time for each cancer type was then estimated by calculating a weighted average of the sojourn types by stage (Table 3).

Table 3: Estimations of sojourn time by stage at diagnosis based on Schwartzberg, et al., 2022 [14], and average overall sojourn time based on current stage distribution at diagnosis.

| **Cancer Type** | **Sojourn Time Stage I** | **Sojourn Time Stage II** | **Sojourn Time Stage III** | **Sojourn Time Stage IV** | **Average Sojourn Time** |
| --- | --- | --- | --- | --- | --- |
| Kidney | 2 years | 5.3 years | 7.4 years | 10.5 years | 5.78 years |
| Liver | 1 year | 2.25 years | 2.75 years | 3.5 years | 2.83 years |
| Stomach | 1.5 years | 4 years | 5.25 years | 6 years | 5.15 years |
| Oesophageal | 1.25 years | 2.75 years | 3.25 years | 4 years | 4.67 years |
| Pancreatic | 0.25 years | 0.75 years | 1.25 years | 2 years | 2.26 years |
| Colon | 1.75 years | 5 years | 6.75 years | 7.5 years | 6.29 years |
| Ureteric* | 1.5 years | 4 years | 5.25 years | 6 years | 5.76 years |
| Gallbladder | 1 year | 2.25 years | 2.75 years | 3.5 years | 2.43 years |
| Adrenal** | 2.5 years | 6.5 years | 9 years | 10.5 years | 6.68 years |
| Hodgkin*** | 2 years | 5.25 years | 7.5 years | 9 years | 6.50 years |
| Non-Hodgkin*** | 2 years | 5.25 years | 7.5 years | 9 years | 7.13 years |
| * Values for urothelial cancer used. **No dwell time data available, so assumed same as kidney cancer; ***Data for lymphomas was not available from Schwartzberg, et al. [14] but was available in a published abstract from the same authors [15]. | | | | | |

For most cancers, cancer stage data was available from NDRS [16], with 2019 data being selected to avoid discrepancies due to COVID-19. There were a small number of cancers for which data was not publicly available from NDRS, and so rapid searching was used to find alternative sources. For liver cancer, stage distribution was provided by ONS survival data counts for each stage – this data was slightly older (2013-17) [17]. For adrenal and gallbladder cancers (both rare cancers), no UK stage data could be identified and instead US sources were used [18, 19]. Stage distribution data can be found in Table 7. NDRS data included a substantial proportion of cancers with missing stage data. There is evidence suggesting that survival in people with missing stage data is very poor in the short-term, but from year one onwards after diagnosis is slightly better than in people with stage IV cancer [20]. In the model it was therefore assumed that cancers with missing stage data would be distributed between stage III and IV in the same ratio as the recorded stage III/IV cancers.

Sojourn times were rounded to whole numbers, and cumulative incidence rate data offset by an equivalent number of years to estimate total prevalence rates (e.g. a sojourn time of 5 years would mean that cumulative prevalence in a population aged 55 would be estimated to be equivalent to cumulative incidence in a population aged 60). These estimates would include both diagnosed and undiagnosed prevalence, so to estimate undiagnosed prevalence specifically, the cumulative incidence for each age was subtracted from the total prevalence estimates for each age. Figure 5 shows the estimated total undiagnosed prevalence rates by age and sex for each cancer, for the target population.

Figure 5: Estimated undiagnosed prevalence rates per 100,000 by age and sex in the target screening population for some of the modelled cancers.


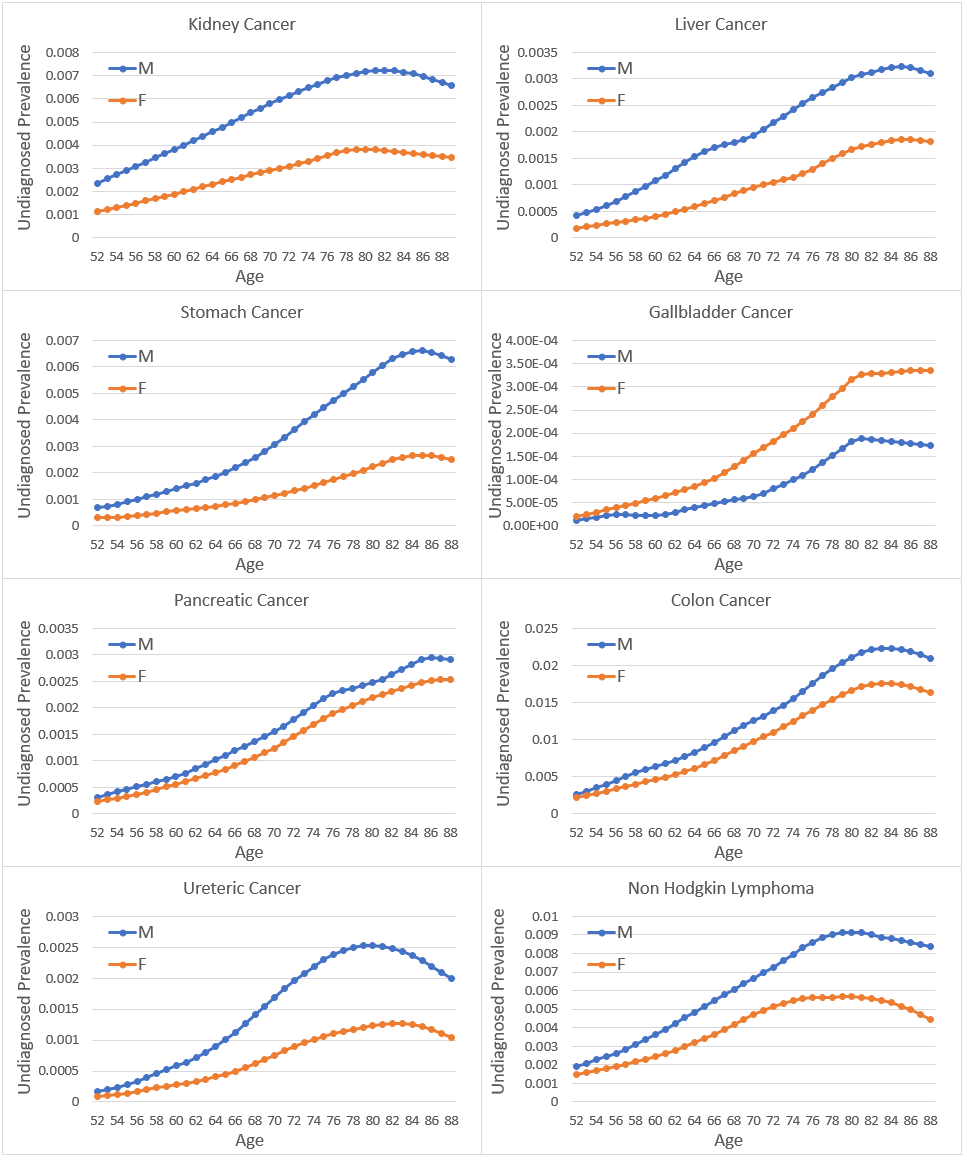


### Estimating screen-detectable cancer prevalence

Not all cancer is detectable specifically through UA screening. This is due to several reasons:

1. CT does not have perfect sensitivity for cancer, with sensitivity varying by organ;
2. The UA scan may not cover the entirety of each organ in all people;
3. The lung scan may cover some of the organ and therefore be able to detect a proportion of cancer;
4. Cancer may not be evenly distributed within an organ, which must be taken into account if b) and/or c) above are true.

There is some literature available to inform the sensitivity of CT scan; however, this is of limited use as it tends not to be assessed in a screening setting, usually assesses other types of CT such as contrast-enhanced, and rarely assesses the sensitivity for detecting a mass that could be cancer, rather than distinguishing cancer from other abnormalities.

Instead of informing this parameter from the literature, informal advice from the radiologists who took part in YKST was sought. They gave rough estimates of the expected average, lower and upper bounds for sensitivity of the UA scan (as incremental to the lung scan) in each organ/cancer type based on the four points outlined above, with mean values assumed equidistant between these (Table 4). Screening sensitivity will vary by cancer stage as larger cancers will be detected more easily (as noted by the radiologists); however, for the purposes of the model it was assumed not to vary by stage, or by age or sex.

Table 4: Estimates of screening sensitivity for different cancer types from YKST radiologists, based on CT sensitivity and anatomical coverage.

| **Cancer/organ** | **Mean** | **Range** | **Justification** |
| --- | --- | --- | --- |
| Kidney | 0.6 | 0.5-0.7 | Good CT sensitivity apart from some small tumours, complete coverage by UA scan but also 25-30% coverage by lung scan. |
| Liver | 0.5 | 0.4-0.6 | Similar coverage to kidney, but lower CT sensitivity due to lack of contrast and most small liver cancers are endophytic. |
| Stomach | 0.1 | 0.05-0.15 | Good UA-specific coverage but CT sensitivity is low unless cancers are advanced. |
| Oesophageal | 0 | 0 | Anything found on UA scan would also be found on lung scan. |
| Pancreatic | 0.5 | 0.4-0.6 | Good coverage but CT sensitivity is low for small tumours. |
| Colon | 0.05 | 0.0-0.1 | CT sensitivity moderate. Only part of the colon seen on UA scan, with most cancers found in locations not covered. |
| Ureteric | 0.75 | 0.6-0.9 | Good coverage of most of target area. CT sensitivity low to small tumours, but will be found if cause hydronephrosis. |
| Gallbladder | 0.5 | 0.4-0.6 | Good coverage by UA scan and unlikely to be seen on lung scan, but sensitivity is fairly low. |
| Adrenal | 0.28 | 0.23-0.33 | Good CT sensitivity but most found on lung scan too. Value used here based on YKST adrenal adenoma findings. |
| Hodgkin | 0.25 | 0.15-0.35 | Fairly good CT sensitivity but only a low proportion present in abdominal lymph nodes or spleen. |
| Non-Hodgkin | 0.066 | 0.034-0.1 | Fairly good CT sensitivity but few exclusively present in abdominal lymph nodes or spleen covered by UA scan. |

Screening sensitivity was combined with estimates of undiagnosed cancer prevalence to produce estimates of screen-detectable cancer prevalence. The number of cancer cases estimated for the YKST population composition is shown in Table 5, together with the number of cancer cases actually observed, and the expected confidence intervals around the observed data based on the sample size. Model estimates were found to be within YKST observed confidence intervals.

Table 5: Comparison of calculated number of cancer cases for the YKST population (deterministic model) versus actual cancer cases observed specifically in the UA scan, and the expected 95% confidence intervals around the observed cases based on the YKST sample size.

| **Cancer/organ** | **Model estimated** | **YKST observed** | **YKST confidence interval** |
| --- | --- | --- | --- |
| Kidney | 10.11 | 10 | 4.8-17.1 |
| Liver | 2.85 | 2 | 0.24-5.6 |
| Stomach | 0.87 | 0 | NA |
| Oesophageal | 0 | 1 | 0.025-3.7 |
| Pancreatic | 2.67 | 1 | 0.025-3.7 |
| Colon | 2.12 | 1 | 0.025-3.7 |
| Ureteric | 3.39 | 2 | 0.24-5.6 |
| Gallbladder | 0.20 | 0 | NA |
| Adrenal | 0.11 | 0 | NA |
| Hodgkin | 0.36 | 1 | 0.025-3.7 |
| Non-Hodgkin | 1.39 | 1 | 0.025-3.7 |
| Metastatic carcinoma* | NA | 1 | 0.025-3.7 |
| *Primary unknown and only primary cancers modelled | | | |

## Estimation of cancer stage distribution at baseline

The effectiveness of cancer screening is driven primarily by an expected shift in the stage distribution at diagnosis in the screening versus comparator arms. However, the YKST only provided a small amount of data to inform stage distribution at screening for kidney cancer with high uncertainty (N = 10), and no data to inform this for other cancer types. A rapid review identified that some published data is available to inform stage distribution at screening for a small number of cancer types; however most of this is from settings that differ quite considerably from England (e.g. the Far East for gastric cancer screening). The exception is for kidney cancer, where a meta-analysis is available [21], the findings of which align closely with the YKST results (Table 6).

To provide a consistent estimate of stage distribution at screening across the cancers included in the model, we developed a method to calculate it based on known stage distribution at diagnosis in current care, and the dwell time data, both of which are described above as part of the prevalence calculations. The same assumptions were made as above regarding missing stage data, and the assumption that people are diagnosed on average halfway through a stage. The proportions of time expected to be spent undiagnosed in each stage were calculated for each stage at diagnosis in current care, and then these proportions normalised to one to obtain the expected stage distribution at screening.

This method does not take into account increasing incidence/prevalence with age, and therefore a) makes the assumption that stage distribution does not differ by age (or sex); b) will slightly underestimate the proportions in early stage disease compared to late stage disease. An alternative method estimating prevalence by stage and age would avoid this, and should be considered in future iterations of the model, but was not implemented here due to lack of time. Note also that stage distribution at screening is likely to be affected by differential screening sensitivity by stage, which was also not implemented in this version of the model. This is likely to have the opposite impact and overestimate the proportion of early stage detected compared with late stage. This should also be considered in future iterations of the model.

Comparison of the calculations for kidney cancer with the YKST and meta-analysis estimates suggested that this approach resulted in a fairly accurate estimate of screening stage distribution (Table 6), and therefore it was applied across all cancer types (Table 7).

Table 6: Kidney cancer stage distribution at screening estimated by the YKST study, the Rossi meta-analysis [21], and through calculations.

| **Stage at diagnosis** | **YKST (N = 10)** | **Meta-analysis** | **Calculations** |
| --- | --- | --- | --- |
| Stage I | 80% | 84% | 57% |
| Stage II | 0% |  | 29% |
| Stage III | 10% | 14% | 12% |
| Stage IV | 10% | 2% | 3% |

Table 7: Stage distribution at diagnosis in screening and comparator arms. Stage distribution in the comparator arm is based on data about current stage distribution at diagnosis from various sources. It was assumed for model purposes that missing stage data would be distributed across stage III and stage IV in the same proportions as the known stage III to stage IV ratio. Stage distribution in the screening arm is calculated using methods given in section ‎3.2..

| **Cancer** | **Arm** | **Stage I** | **Stage II** | **Stage III** | **Stage IV** | **Missing** | **Source** |
| --- | --- | --- | --- | --- | --- | --- | --- |
| Kidney | Comparator | 34.1% | 5.1% | 14.3% | 15.8% | 30.6% | [16] |
|  | Screening | 56.6% | 28.8% | 11.9% | 2.7% |  |  |
| Liver | Comparator | 13.1% | 15.5% | 13.9% | 57.5% | NA | [17] |
|  | Screening | 55.1% | 23.3% | 11.4% | 10.2% |  |  |
| Stomach | Comparator | 10.0% | 10.6% | 11.9% | 29.5% | 38.0% | [16] |
|  | Screening | 59.9% | 24.0% | 10.7% | 5.3% |  |  |
| Oesophagus | Comparator | 5.1% | 9.3% | 22.8% | 35.6% | 27.1% | [16] |
|  | Screening | 66.0% | 16.1% | 12.3% | 5.6% |  |  |
| Pancreas | Comparator | 7.3% | 8.5% | 10.1% | 43.5% | 30.6% | [16] |
|  | Screening | 35.4% | 32.6% | 16.8% | 15.1% |  |  |
| Colon | Comparator | 13.5% | 23.4% | 23.0% | 20.6% | 19.5% | [16] |
|  | Screening | 51.9% | 39.6% | 6.2% | 2.4% |  |  |
| Ureteric | Comparator | 34.1% | 5.1% | 14.3% | 15.8% | 30.6% | [16] |
|  | Screening | 55.6% | 30.1% | 11.6% | 2.7% |  |  |
| Gallbladder | Comparator | 11.3% | 29.6% | 12.7% | 42.2% | 4.1% | [18] |
|  | Screening | 64.7% | 15.2% | 10.8% | 9.3% |  |  |
| Adrenal | Comparator | 14.5% | 14.5% | 35.5% | 35.5% | NA | [19] |
|  | Screening | 54.8% | 30.8% | 11.8% | 2.6% |  |  |
| Hodgkin Lymphoma | Comparator | 10.2% | 24.9% | 18.1% | 23.2% | 23.7% | [16] |
|  | Screening | 58.4% | 25.8% | 13.0% | 2.8% |  |  |
| Non-Hodgkin Lymphoma | Comparator | 10.6% | 7.6% | 13.6% | 38.8% | 29.5% | [16] |
|  | Screening | 53.1% | 26.0% | 16.6% | 4.2% |  |  |

## Estimation of screen-detectable prevalence & size of AAA at baseline

Data directly from the YKST study was used to inform screen-detectable prevalence and size of AAA at baseline. This was possible due to the relatively large numbers of AAA found in the study (N = 60). AAA findings in YKST were much greater in men than women, and also showed an increasing prevalence with age. Only AAAs which were new findings (i.e. not previously known through the AAA screening programme) and were seen only on the abdominal scan (i.e. not visible on the lung health check scan) were included.

An exponential model for all sizes of AAA and both sexes was fitted to the trial prevalence data using the form log(prevalence) = fx(age). The coefficients and error terms for this model are shown in Table 8. Due to the small numbers of AAA observed for each age group in YKST, the exponential model resulted in inappropriately high levels of uncertainty when estimating total AAA prevalence; therefore the standard errors were manually calibrated to give 95% confidence intervals which corresponded to the observed uncertainty in YKST. The predicted values from this model are shown alongside the trial prevalence data in Figure 6.

Table 8: Parameters for an exponential model of AAA prevalence by age

|  | Estimate | Std. Error | Adjusted Std. Error |
| --- | --- | --- | --- |
| Intercept | -8.389 | 0.247 | 0.0633 |
| Age | 0.0601 | 0.00354 | 0.000908 |

Figure 6: YKST data (purple) and exponential model predictions (blue) of AAA prevalence by age


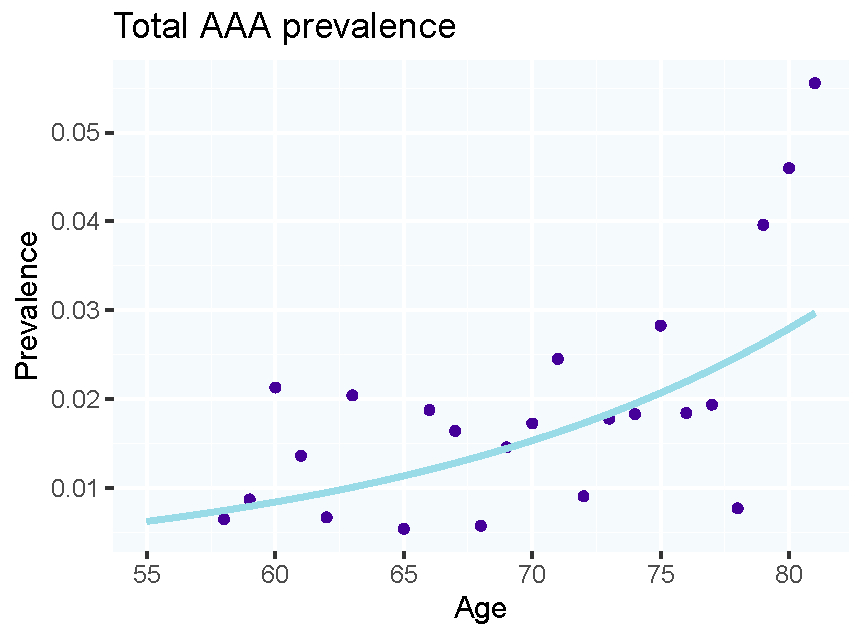


Prevalence for males and females was estimated from total prevalence based on the assumption that the ratio of male to female cases did not vary by age. A similar approach was used to assign the three sizes of AAA ((3.0-4.4cm; 4.5-5.4cm; and >5.4cm). Beta distributions for these estimates based on YKST data are shown in Table 9.

Table 9: Parameters used to estimate AAA prevalence by size and sex

| Parameter | Proportion | alpha | beta |
| --- | --- | --- | --- |
| Male | 0.8 | 48 | 12 |
| Female | 0.2 | 12 | 48 |
| Small | 0.77 | 46 | 14 |
| Medium | 0.18 | 11 | 49 |
| Large | 0.05 | 3 | 57 |

This approach resulted in an estimation of 60.3 AAA cases in total for the modelled YKST population; comparable to the 60 cases actually found in the study.

## Estimation of screen-detectable prevalence of incidental findings at baseline

The prevalence of incidental findings was implemented in the model as a ratio of incidental findings to cancers for the same target organ. Deterministic model estimates of YKST cancer prevalence (see Table 5) were used as model inputs for the cancer side of the equation, whilst data from YKST was used to inform incidental finding prevalence (Table 10). This was possible because for most organs, the number of incidental findings was much higher than the number of cancers found and so was likely to be fairly robust. People with more than one finding were excluded for analysis, as were people with findings that were known previously or found in the lung scan.

Table 10: The number of incidental findings for each target organ found in the YKST UA screen, and the calculated ratios based on dividing number of incidental findings by modelled estimates of cancer cases in YKST (Table 5).

| **Cancer/organ** | **Number incidental findings** | **Ratio incidental findings to cancer** |
| --- | --- | --- |
| Kidney | 1063 | 105.1 |
| Liver | 16 | 5.6 |
| Stomach | 0 | 0 |
| Oesophagus | 0 | 0 |
| Pancreas | 30 | 11.2 |
| Colon | 3 | 1.4 |
| Ureteric System | 3 | 0.9 |
| Gallbladder | 16 | 78 |
| Adrenal Gland | 45 | 411 |
| Lymph System | 2 | 2.8 (Hodgkin); 0.7 (Non-Hodgkin) |

Whilst the study was insufficiently large to determine whether there may be differences in rates of incidental findings by age and sex, there is likely to be a general trend whereby incidental findings increase with age, as this is true for the prevalence of many of the conditions identified. Using a ratio approach thereby enabled prevalence of modelled incidental findings to vary by age and sex in the same way as cancer findings, by multiplying the estimated ratio by the modelled number of cancers for a particular age/sex cohort. If a particular target organ had no incidental findings, the ratio was assumed to be zero.

# Markov model health states and transitions

Three types of Markov models are included in the health economic model, those for cancer, those for AAA and those for no disease. The ‘no disease’ model was also used to model long-term outcomes in the proportion of the cohort with incidental findings. All Markov models were designed with annual cycles and lifetime horizons, and were half-cycle corrected to account for transitions occurring on average halfway through the year.

## No disease Markov models

### Structure

The no disease Markov model has two health states: alive and dead from all causes (Figure 7), with the whole cohort starting in the alive state at model start. The model is identical for the screening and comparator arms. There are no costs or utility decrements attached to the no disease Markov model. There is a single transition from alive to dead based on the all-cause mortality data described in section ‎4.4 below.

Figure 7: The structure of the no disease Markov model


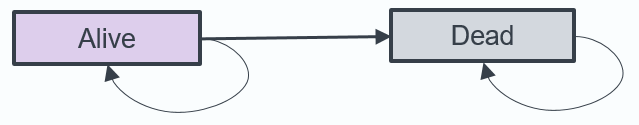


## Cancer Markov models

### Structure

The long-term Markov models for each of the cancer types were designed with an identical structure (Figure 8). The models include three sets of health states for each cancer stage; 1) undiagnosed; 2) ‘year one diagnosed’; 3) ‘subsequent years diagnosed’. This structure enables different costs, utility decrements and survival to be implemented in the first and subsequent years after diagnosis, which is important to represent the impacts of a cancer diagnosis, which are particularly high in the first year. There is also a cancer death health state and an other-cause mortality health state.

In the screening arm of the model, the entire cohort starts in the ‘year one diagnosed’ health states based on the calculated stage distribution at screening. In the comparator arm of the model, the entire cohort is similarly distributed into stages at model start, but in the equivalent undiagnosed health states. There are three types of transition probabilities in the cancer Markov models; stage progression, diagnosis and mortality.

Figure 8: The structure of the cancer Markov models


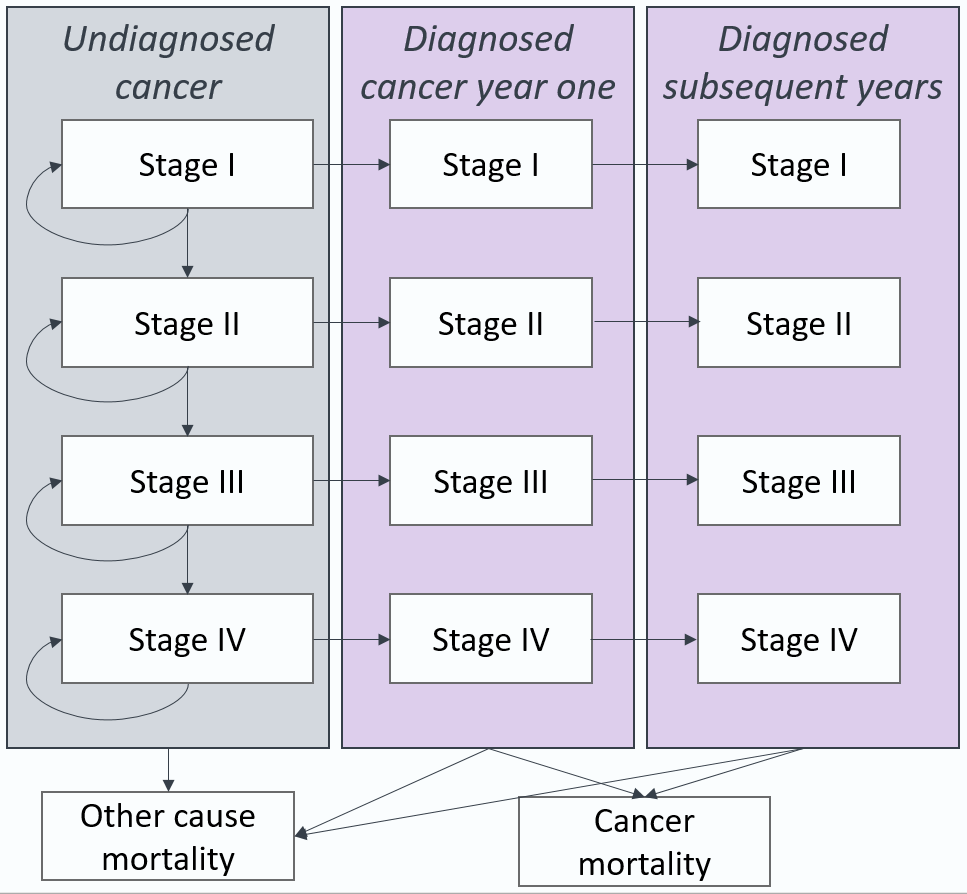


### Cancer-specific mortality

All living health states have an annual probability of transition to death from either all causes (if undiagnosed) or other causes (if diagnosed with cancer), based on the data described in section ‎4.4 below. Those diagnosed with cancer also have a probability of cancer-specific mortality.

Cancer mortality was based upon one-year and five-year net survival by age group, sex & stage at diagnosis for those diagnosed between 2013 and 2017 in England, from the Office for National Statistics (ONS) [17]. Some fields had survival estimates of greater than 100%, which were adjusted to 100%. For some cancers there was missing data for one of the sexes, some age groups or some stages at one or more time points, and for a small minority of cancers there was no data available at all. A series of rules were applied in the following order to fill in missing data, which prioritised stage differences over age differences, and age differences over sex differences:

1. If data was missing for males or females, but present for persons, missing data was replaced with persons data.
2. Missing data for age groups <55 (used only for interpolation) was replaced with data from the age 55-64 group.
3. If data for some stages was present in some >55 age groups but missing in others, missing data was estimated as: all ages value for that stage * specific age value for all stages/all ages value for all stages.
4. If data from some stages was missing for all age groups, the all ages value was used for all age groups.
5. If no stage-specific data was available, this was estimated based on data (by age group, stage & sex) from a similar cancer type * specific age value for all stages for desired cancer type/specific age value for all stages for similar cancer type. This applied to pancreatic cancer (for which data from stomach cancer was applied), ureteric cancer (for which data from kidney cancer was applied), and non-Hodgkin lymphoma (for which data from Hodgkin lymphoma was applied).
6. No data was available at all for adrenal or gallbladder cancer. Adrenal cancer was assumed to have the same survival as kidney cancer, whilst gallbladder cancer was assumed to have the same survival as liver cancer.

Mortality in the ‘year one diagnosed’ health states was calculated as the inverse of the year one survival data. Annual mortality in the ‘subsequent years diagnosed’ health states was assumed to be equivalent to the annual probability of death between year one and year five after cancer diagnosis. Uncertainty in the survival data was incorporated in the model. To ensure that subsequent year mortality would not inadvertently end up being negative through sampling of lower one-year than five-year survival in uncertainty analysis, the two sets of distributions were assumed to be perfectly correlated. Age group mortality data was interpolated to provide values for each age by sex.

There were several limitations around the way the data was used, caused by the lack of diagnostic history in the model - a consequence of the Markov structure. Firstly, mortality probabilities were applied based on current age in the model, whereas they actually relate to age at diagnosis. Secondly, a constant probability of cancer death was applied to all subsequent years after diagnosis, and it is unclear whether there is any significant cancer death more than ten years after diagnosis. Model validation confirmed that overall mortality ten years after diagnosis was similar to published estimates, but both limitations may mean that ‘lifetime’ death from cancer is slightly overestimated, particularly in younger cohorts who have longer to live. There is no data to validate this against, so it is unclear how large the impact may be.

It was also assumed that those with undiagnosed cancer would have no probability of cancer-specific mortality, given that the survival data is based only on diagnosed cases. This does mean that cohorts diagnosed earlier through screening initially have higher cancer-specific mortality than those who have a delay in diagnosis in the comparator arm, and this is only overcome later in time by stage shift mortality benefits if they are significant enough. Note that this is the inverse of the lead time bias that affects trial data; there is no lead time bias in the model so potentially mortality could be overestimated in the screening arm. It is unclear what would actually happen in practise, but some screening trials for other cancer types have found no mortality benefits despite stage shift [22, 23], suggesting that this modelling assumption might not be completely implausible. There is also some evidence that stage-specific survival could be significantly higher in screen-detected compared with symptomatic-detected colorectal cancer patients [24], although it is unclear whether at least some of this might be due to differences in patient characteristics amongst those who are eligible and decide to attend screening (e.g. age, socioeconomic status). Given the uncertainty in that data, the basecase model analysis assumed stage-specific mortality did not change between arms, but sensitivity analysis was carried out where mortality was reduced in the screening arm compared with the comparator arm, in line with the colorectal cancer data [24].

### Stage Progression

There is a probability of progression from undiagnosed cancer stages I-III to the next stage. Diagnosed cancer stages were assumed not to progress, and progression/recurrence was not modelled explicitly as a separate health state. This is because the cancer specific mortality data described above is based on stage at diagnosis, and the risk of progression/recurrence is implicitly reflected in that data as a stage-dependent probability of cancer mortality. In order to incorporate the impacts of post-diagnosis progression/recurrence in the model, it was assumed for cost/utility purposes that it occurred in the year before cancer death.

Modelled stage progression for undiagnosed cancer was based on the dwell time data described in section ‎3.1. A set of tunnel states was set up corresponding to each whole or part year spent in each stage according to the dwell time data. The population in each stage at model start was divided proportionally between the relevant tunnel states, with any tunnel states reflecting part of a year receiving a corresponding fraction of the population. Cohorts of each tunnel state were assumed to transition completely to the next tunnel state each year, unless they were diagnosed or died from all-cause mortality. This meant that each stage transition probability would increase over time as the distribution of the population in that stage diminished, culminating in 100% transition as the last proportion of the cohort moved into the next stage. Stage transition probabilities also differ by age and sex due to corresponding differences in all-cause mortality.

### Symptomatic diagnosis

The annual probability of symptomatic diagnosis from undiagnosed cancer stages I-III was calculated through a simple iterative calibration process using the comparator arm of the cancer Markov models (Figure 9). Given the lack of dwell time data for stage IV it was assumed that all people in stage IV would be diagnosed within a year. The aim of the calibration was to run the Markov models from the start, where the cohort is distributed in undiagnosed stages, until they matched the known current care stage distributions in the diagnosed states. To simplify this process, death after diagnosis (from any cause) was excluded from the model, although death prior to diagnosis from all causes was retained as this would alter the values calibrated.

The model was run initially with symptomatic diagnosis happening only from stage IV. A set of starting symptomatic diagnosis parameters for the other stages was then estimated by dividing the known current care stage distributions at diagnosis by the proportion of the population present across all years of the tunnel states for each stage in the preliminary Markov trace. The model was run and then the symptomatic diagnosis parameters were updated using a multiplier derived from the difference between the expected diagnosed stage distribution and the one modelled in that iteration.

Modelled diagnosed stage distributions generally matched the known stage distribution data within ten iterations. Probabilities of symptomatic diagnosis by stage calibrated through this method are shown in Table 11. It would be expected that the symptomatic diagnosis rate might increase with advancing stage, as larger and more serious cancers would in general be expected to cause more symptoms. This is not predicted consistently by the calibration, suggesting that estimates of dwell time may be incorrect and incompatible with the known stage distribution at diagnosis. For kidney cancer, this is particularly apparent for stage II. Few people are diagnosed at stage II currently, but the estimated dwell time is relatively long (median of 3 years), resulting in a much lower annual probability of symptomatic diagnosis compared with stage I. However, these discrepancies are unlikely to have a significant impact on the model results, particularly given that the model estimates of kidney cancer prevalence and screening stage distribution (both of which also use dwell time data – see Section ‎3 above) reproduce the YKST study fairly well.

Table 11: Calibrated annual probabilities of symptomatic diagnosis by cancer stage

| **Cancer** | **Stage I** | **Stage II** | **Stage III** |
| --- | --- | --- | --- |
| Kidney | 0.300 | 0.042 | 0.365 |
| Liver | 0.168 | 0.246 | 0.341 |
| Stomach | 0.073 | 0.095 | 0.316 |
| Oesophageal | 0.035 | 0.117 | 0.461 |
| Pancreatic | 0.195 | 0.144 | 0.337 |
| Colon | 0.113 | 0.114 | 0.549 |
| Ureteric | 0.307 | 0.040 | 0.365 |
| Gallbladder | 0.122 | 0.595 | 0.322 |
| Adrenal | 0.106 | 0.087 | 0.383 |
| Hodgkin Lymphoma | 0.067 | 0.175 | 0.305 |
| Non-Hodgkin Lymphoma | 0.076 | 0.053 | 0.171 |

Figure 9: Calibration and Markov component of model

## AAA Markov models

### Structure

The long-term Markov model for AAA was based upon a pre-existing model that assessed the cost-effectiveness of the English NHS AAA screening programme [25]. The model structure was simplified to minimise the number of health states required (Figure 10). The adapted model includes three undiagnosed health states in line with the classification of AAA data into three size groups, in addition to small and medium size AAA diagnosed health states, which are each associated with a particular frequency of surveillance. It is assumed that anyone diagnosed with large AAA will move directly to elective surgery and then post-surgery survival or death within the same annual cycle, so there is no health state specifically for diagnosed large AAA. Note that this is a simplification from the published model structure whereby some people may be contraindicated for surgery, and some other large AAAs may be returned to surveillance for a while rather than operated on immediately.

All AAA health states have a risk of rupture, of which a proportion will undergo emergency surgery and a proportion of those will survive, with all other ruptures resulting in death. Given that costs of surgery fall into a single year only, and these costs differ between elective and emergency surgery, there are four ‘year one’ post-surgery health states (elective vs emergency, survive vs die), which are tunnel states leading into ‘subsequent year’ health states representing both ongoing survival and death post-surgery. There is also an other-cause mortality health state.

In the screening arm of the model, the entire cohort starts in either diagnosed small AAA, diagnosed medium AAA, or elective surgery ‘year one’ health states, based on the modelled size distribution at screening. In the comparator arm of the model, the entire cohort is similarly distributed into the three undiagnosed size states at model start.

Figure 10: Structure of the AAA Markov model


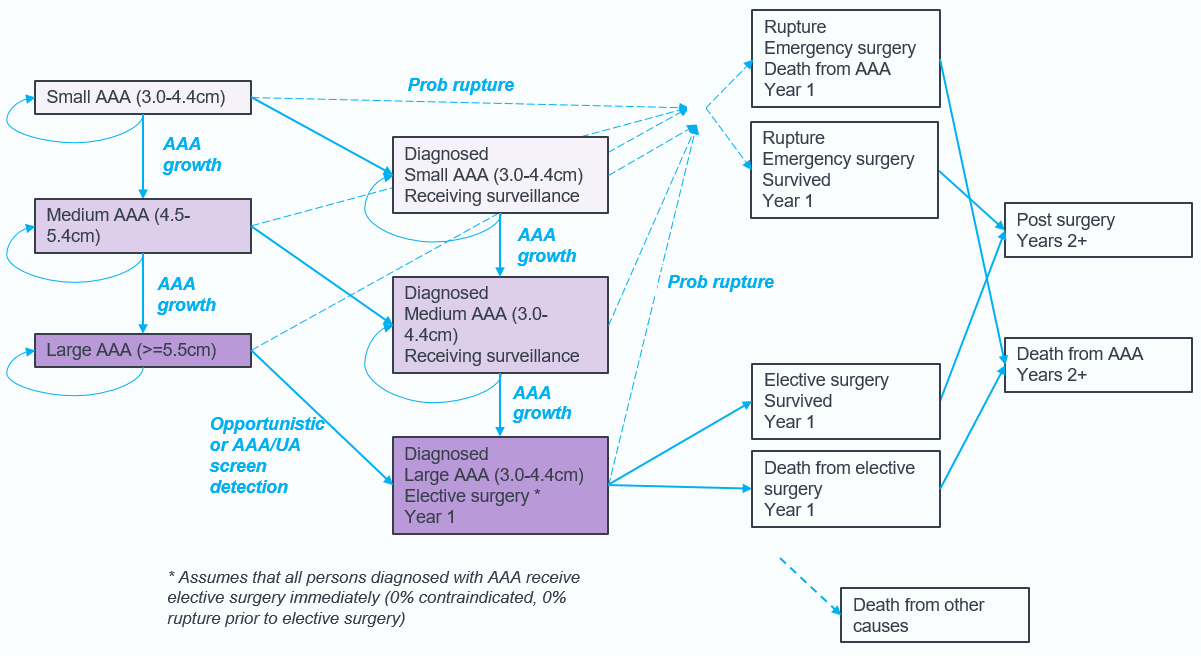


### Growth, rupture, diagnosis and death

Transition probabilities for AA growth, rupture and opportunistic diagnosis were taken directly from the English NHS AAA screening programme model [25] (Table 12). Note that there is some evidence that there may be differences in AAA growth and rupture by sex [26]; however, for simplicity it was assumed that the same transition probabilities could be applied across all ages and both sexes. Mean values were used where growth and rupture probabilities were time-dependent in the original model. All transition probabilities were converted from three-month to annual cycles via the standard rate conversion equation.

Table 12: Transition probabilities used for AAA growth, rupture and diagnosis, taken from the English NHS AAA screening programme model[25].

| **Transition** | **Published 3-month Transition Probability** | **Applied Annual Transition Probability** |
| --- | --- | --- |
| Growth: small to medium AAA | 0.016 | 0.062 |
| Growth: medium to large AAA | 0.077 | 0.274 |
| Rupture: small AAA | 0.00076 | 0.00303 |
| Rupture: medium AAA | 0.0064 | 0.025 |
| Rupture: large AAA | 0.0282 | 0.108 |
| Diagnosis: opportunistic | 0.0114 | 0.0045 |
| Diagnosis: screening (men aged 65 only) | NA | 0.741* |
| *Based on uptake of 0.75 and sensitivity of 0.988 from [25]. | | |

In addition to opportunistic diagnosis, the existence of the AAA screening programme means that men aged from 65 are eligible for a one-off ultrasound screen. It was important to include this source of diagnosis in the current care arm of the model. For simplicity it was assumed that men would only attend screening at age 65 (and not above this age), with an uptake of 75% and sensitivity for AAA detection of 98.8% based on the English NHS AAA screening programme [25].

It was assumed that everyone diagnosed with large AAA (whether moving from the undiagnosed health state, from the diagnosed medium AAA health state, or diagnosed through screening at model start) would undergo elective surgery within the same year and either survive or die. In contrast, only a proportion of ruptured AAA would undergo emergency surgery, and only a proportion of those would survive. Proportions undergoing surgery and dying were obtained from the English NHS AAA screening programme model [25] (Table 13). A weighted average of values from screen-detected and opportunistically detected AAA cases was used to estimate the proportion dying after elective surgery.

Table 13: Proportions of the population undergoing surgery and dying from surgery for AAA

| **Type of Surgery** | **Proportion undergoing Surgery** | **Proportion Dying after Surgery** |
| --- | --- | --- |
| Elective Surgery (diagnosed large AAA) | 1* | 0.042 |
| Emergency Surgery (ruptured AAA) | 0.368** | 0.342 |
| *Assumption; **Note that the remainder who do not undergo emergency surgery are assumed to die | | |

## All/other cause mortality

Probabilities for all-cause mortality by age and sex were obtained from ONS English life tables for 2018-2020 [27]. More recent data is available, but this was not used in order to avoid incorporating the excess death rates caused by COVID-19. Other-cause mortality was estimated separately for each modelled disease by determining the proportion of all registered deaths that were due to each disease, for each age and sex, using death registration summary statistics for England and Wales (2021) [28]. Other cause mortality for each disease was calculated separately by subtracting disease-specific mortality from all-cause mortality using the following equation:

Oth_Cause_Mort_(age, sex)_ = All_Cause_Mort_(age, sex)_ * (1 – (N_Disease_Deaths_(age, sex)_ / N_All_Deaths_(age, sex)_))

It was assumed that the probability of all or other-cause mortality would be 100% in populations aged above 100.

Populations with smoking history have a higher risk of many different diseases and correspondingly higher risk of all cause death. The disease explicitly modelled will only represent a small proportion of the increased risk that smokers have, so additional steps were taken to reflect this risk in the modelled population. This was implemented in the modelling by applying a relative risk uniformly across the all and other-cause mortality data for all ages and sexes. The relative risk was calculated based on the proportion of current to former smokers eligible for YKST, and data taken from a UK prospective study of 1 million women [29]. This study estimated a relative risk for all cause mortality of 2.76 for current smokers and 1.56 for former smokers who had stopped smoking at age 45-55. Data from this group was used rather than from groups who stopped smoking at younger ages as only the highest risk former smokers are eligible for YKST (risk is based partly on time since stopping smoking). No equivalent data was found for men, and therefore the same relative risks were applied for both sexes.

# Resource use, cost and utility inputs

The resource use, unit cost and utility inputs used in the model are described below. Costs took an NHS perspective and were inflated to 2022 values where necessary using the NHS Pay and Prices cost inflation index [30]. Model output costs and QALYs were discounted at 3.5% in the basecase scenario, in line with NICE guidelines [31].

## Resource use and costs

### Screening pathway

Screening resource use and costs were determined with help from the YKST team who detailed the screening pathway undertaken for people with negative and positive results in the YKST study. The aim was to estimate the likely cost of upper abdominal screening in practice as an add-on to the existing lung checks, using the YKST resource use as a guide. This meant that resource was only included if it was: a) incremental to use of resources in the lung scan; b) was specifically required for the clinical pathway rather than e.g. as part of study administration; c) would be expected to be required in any future roll-out of screening in practice. The full screening pathway is shown in Figure 11.

Figure 11: The YKST screening pathway


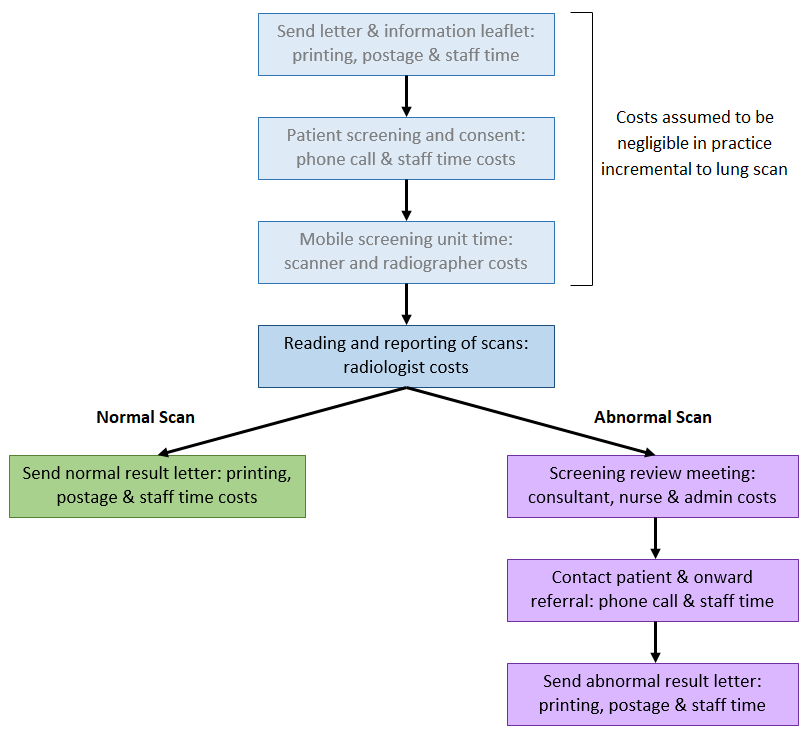


Several elements of the pathway that were carried out for YKST were considered unlikely to be required for screening in practice when considered incrementally to the lung checks, and were not included in the screening costs. So for example, whereas YKST involved separate distribution of informational material and consenting of patients, it was thought that in practice this would be combined with the existing lung information and consenting process thereby resulting in no additional cost. Similarly, radiologist input indicated that the additional scan itself would take only a second or two to perform and therefore would not require any additional time on the mobile scanning unit. However, lung and abdominal scans required sending separately to different specialist radiology teams for interpretation and reporting, therefore this represented an additional cost, as did clinical review meetings for positive results, and informing patients of their results.

All resource use costs were estimated per person screened (See Table 1 of main manuscript). Whilst in YKST a small proportion of people invited to take part declined, the additional costs of inviting these patients was not included in the basecase screening costs as it was considered that this would not represent an additional cost above and beyond that already incurred by the lung scan if carried out in practice. The cost of a positive result is greater than that of a negative result due to clinical review to determine whether further investigation is required, and telephoning the patient to discuss their results. Whilst in YKST all findings were reviewed clinically, it was thought that in practice 66.6% of non-serious findings (relating to small kidney stones and cysts) would not require clinical review and therefore screening would cost the same for them as for negative results.

Staff costs for clinical review meetings and for informing patients of their results were calculated based on the cost per working hour (including qualifications) for hospital staff [30]. It was assumed that a screening review meeting lasting one hour would be required to review 15 scans, and be staffed by a consultant, a band 6 clinical nurse specialist and a band 3 admin staff. The latter two staff types were also costed in to inform patients by letter (all patients) and phone call (positive findings only), and if necessary to refer the patients by email to the appropriate team for further investigation. It was assumed that informing patients would take a total of 10 minutes of staff time for a negative result and 15 minutes of staff time for a positive result, divided equally between the clinical nurse and admin staff.

Screening costs were applied only to the screening arm of the model. The lower screening costs were assigned to the cohort with no findings, together with 66.6% of the cohort with non-serious incidental findings (equivalent to 57.6% of all incidental findings), with the higher screening cost applied to the remainder.

### Diagnostic pathways

Diagnostic resource use pathways were based upon data from the UA and lung scans (YKST plus YLST), combined to increase significance. Data for each organ was divided into cancers/AAA and all other incidental findings, and data about the type of resources used and the proportion of people requiring each resource was extracted for each set of diseases by the YKST team. People with more than one finding were excluded for this analysis, as were people with findings that were previously known. To restrict the number of different diagnostic resources included in the model to a manageable level, and to avoid inclusion of resources that might not be a direct consequence of screening, most resources were only included if at least 5% of people with a particular finding used them. However, some costly kidney procedures that occurred in a small number of people were included, as these were known to be the direct consequence of some of the incidental kidney findings. If all people with a particular finding had used a resource in YKST/YLST, it was assumed to be an obligate part of the clinical diagnostic pathway and included in the model without uncertainty, whereas if only a proportion of people with a disease had used a resource, it was parameterised with uncertainty.

The following resource types were included in the model:

- Blood tests (any type);
- Urine tests;
- Contrast enhanced CT scan;
- Magnetic resonance imaging (MRI);
- Ultrasound;
- Endoscopic retrograde cholangiopancreatography (ERCP);
- Electrocardiogram (ECG);
- Colonoscopy;
- Biopsy,
- A set of more complex kidney procedures related to treatment of some incidental findings, including ablation, lithotripsy, stent insertion, and surgery which were grouped together for ease of modelling.

A summary of the proportion of people with each finding requiring each diagnostic resource from YKST/YLST is shown in Table 14. As no stomach, gallbladder or adrenal cancers were found some assumptions were made relating to their diagnostic pathways;

- Diagnostic pathways for stomach cancer were assumed to be the same as for oesophageal cancer;
- Diagnostic pathways for gallbladder cancer were assumed to be the same as for liver cancer.
- Diagnostic pathways for adrenal cancer were assumed to be the same as for kidney cancer, albeit with the inclusion of a urine test in the same proportion of people as seen in adrenal incidental findings.

In the model, diagnostic resources use was assumed to be the same for cancers/AAA whether diagnosed through screening in the screening arm, or through symptomatic/chance detection in the comparator arm. However, resource use for incidental findings was assumed to be incurred only in the screening arm of the model.

Table 14: Summary of diagnostic resources and proportion of people using each resource for each diagnosis based on YKST/YLST data.

| Diagnosis | Blood Tests | Urine Tests | Contrast Enhanced CT | MRI | Ultrasound | ERCP | ECG | Colonoscopy | Biopsy | Treatment kidney findings |
| --- | --- | --- | --- | --- | --- | --- | --- | --- | --- | --- |
| Kidney Cancer | 1 | - | 1 | 0.17 | - | - | - | - | 0.42 | - |
| Kidney:  Incidental Findings | 0.22 | - | 0.16 | - | - | - | - | - | - | 0.01 |
| Liver Cancer | 0.5 | - | 0.5 | 0.5 | - | - | - | - | 1 | - |
| Liver:  Incidental Findings | 0.63 | - | 0.19 | 0.13 | 0.44 | - | - | - | - | - |
| Stomach* Cancer | 1 | - | 1 | - | - | - | - | - | - | - |
| Oesophageal* Cancer | 1 | - | 1 | - | - | - | - | - | - | - |
| Pancreatic Cancer | 1 | - | 1 | - | - | - | - | - | 1 | - |
| Pancreas:  Incidental Findings | 0.31 | - | 0.19 | 0.19 | - | - | - | - | - | - |
| Ureteric Cancer | - | - | 1 | - | - | - | - | - | 0.5 | - |
| Ureter & Bladder**:  Incidental Findings | 0.5 | - | 0.5 | - | 0.5 | - | - | - | - | - |
| Colon Cancer | 1 | - | 1 | 1 | - | - | - | 1 | - | - |
| Colon:  Incidental Findings | 0.5 | - | 0.5 | - | - | - | - | 0.25 | - | - |
| Gallbladder Cancer | 0.5 | - | 0.5 | 0.5 | - | - | - | - | 1 | - |
| Gallbladder:  Incidental Findings | 0.23 | - | 0.08 | 0.08 | 0.23 | 0.23 | - | - | 0.15 | - |
| Adrenal Cancer | 1 | 0.38 | 1 | 0.17 | - | - | - | - | 0.42 | - |
| Adrenal gland:  Incidental Findings | - | 0.38 | 0.11 | - | - | - | - | - | - | - |
| Lymphoma | 0.33 | - | 0.67 | - | - | - | - | - | 0.67 | - |
| Spleen/lymph nodes:  Incidental Findings | 0.5 | - | 0.5 | - | - | - | - | - | - | - |
| AAA* | - | - | 0.05 | - | 0.09 | - | 0.05 | - | - | - |
| *No incidental findings for these organs; **Note includes bladder incidental findings too. | | | | | | | | | | |

In the comparator arm, people with cancer are not found through screening, so were assumed to incur additional ‘identification’ resource use not incurred in the screening arm. This was assumed to include additional GP appointments and emergency presentations. A study of NHS audit data from 2013 was used to inform the average number of GP visits required following symptom development before the patient was referred to secondary care [32]. The average across all cancer types (assuming that 5+ visits was equivalent to 5 visits) was 1.74 GP visits.

Data on the proportion diagnosed through emergency presentation was obtained from the National Cancer Registration Dataset [33]. Values for a year’s worth of data from the third quarter of 2021 to the second quarter of 2022 were averaged (Table 15). Values for both kidney and ureteric cancer were informed through data from cancer of the kidney and unspecified urinary organs, as was adrenal cancer for which no specific data was available. Values for gallbladder cancer were informed through the data for liver cancer.

Table 15: Proportion of cancer cases first presented as an emergency, based on National Cancer Registration Data from Q3 2021 to Q2 2022 [33].

| **Cancer Type** | **Proportion Emergency Presentations** |
| --- | --- |
| Kidney | 15.3% |
| Liver | 46.1% |
| Stomach | 30.4% |
| Oesophageal | 20.3% |
| Pancreatic | 55.5% |
| Colon | 18.5% |
| Ureteric | 15.3% |
| Gallbladder | 46.1%* |
| Adrenal | 15.3%** |
| Hodgkin Lymphoma | 16.4% |
| Non-Hodgkin Lymphoma | 28.6% |
| *Assumed to be the same as for liver cancer; **Assumed to be the same as for kidney cancer | |

For most resource use, costs were taken from the National Cost Collection: National Schedule of NHS Costs 2021/22 [34], based on the average for total Health Resource Groups. The exception was the cost of a GP appointment, which was taken from the PSSRU Unit Costs of Health and Social Care 2022 [30]. Description of costs used for each resource are shown in Table 16. Where multiple codes were available (e.g. for contrast-enhanced CT scan there were multiple codes relating to scanning of different numbers of areas), a weighted average was taken. Only costs for adults aged 19 years and over were included. Different types of biopsy with different unit costs were identified for kidney, liver and abdominal cavity, so these were separated in the model based on the relevant organ (abdominal cavity relating to anything other than kidney or liver). A composite cost was used for the treatment of incidental kidney findings category, based on a weighted average of costs in the proportions found in YKST. Within this composite, costs for ablation, lithotripsy and stent insertion came from the National Schedule of NHS Costs [34], whilst the cost of surgery was assumed to be the same as the first year of cost for stage I kidney cancer (see below).

Table 16: Unit costs used for diagnostic resource use. All costs come from the National Schedule of NHS Costs [34] unless otherwise stated.

| **Diagnostic Resource** | **Unit Cost** | **Assumptions** |
| --- | --- | --- |
| GP appointment | £41 | Per surgery consultation lasting 9.22 minutes, with qualifications & including direct care staff costs* |
| Emergency presentation | £157.60 | Consultant-led emergency medicine service. Service code 180 |
| Blood test | £7.66 | Haematology (DAPS05) plus phlebotomy (DAPS08) |
| Urine test | £1.55 | Clinical Biochemistry (DAPS04) |
| Contrast enhanced CT scan | £197.43 | Weighted average CT scan of one or more areas with contrast (RD21A;RD22Z;RD24Z;RD26Z:RD27Z), plus cost of diagnostic imaging outpatient attendance (812) |
| MRI scan | £298.83 | Weighted average MRI scan of one or more areas with contrast (RD02A; RD03A; RD05A), plus cost of diagnostic imaging outpatient attendance (812) |
| Ultrasound | £116.94 | Weighted average ultrasound scan <20 minutes and 20+ minutes (RD40Z; RD42Z), plus cost of diagnostic imaging outpatient attendance (812) |
| ECRP | £239.27 | ECRP without biopsy (GB11Z) , plus cost of diagnostic imaging outpatient attendance (812) |
| ECG | £226.90 | ECG (EY51Z) , plus cost of diagnostic imaging outpatient attendance (812) |
| Colonoscopy | £718.92 | Diagnostic colonoscopy with biopsy (FE31Z), plus cost of histology/histopathology (DAPS02) |
| Biopsy: Abdominal cavity | £810.47 | Percutaneous biopsy of abdominal cavity (YF05Z) plus cost of histology/histopathology (DAPS02) |
| Biopsy: Liver | £365.95 | Weighted average of liver biopsy (YG10Z; YG11A) plus cost of histology/histopathology (DAPS02) |
| Biopsy: Kidney | £390.06 | Needle biopsy of kidney lesion (YL20A) plus cost of histology/histopathology (DAPS02) |
| Treatment of incidental kidney findings | £3,625.24 | Weighted average of surgery**, lithotripsy (LB36Z), kidney stent (YL11Z) and ablation (YL01Z; YL02Z). |
| *From PSSRU Unit Costs of Health & Social Care 2022 [30]; **From cost of year 1 treatment of stage I kidney cancer (see below). | | |

### Cancer treatment

There is a lack of detailed and consistent data across cancer types about costs of cancer treatment, and in particular the difference in costs by cancer stage at diagnosis, which is particularly important to represent when the model benefits are driven by stage shift. There are detailed costs available for kidney cancer from the Rossi model [35], and given that this was the most prevalent cancer type in the model, for consistency an assumption was made that treatment of other cancer types would incur the same unit costs as kidney cancer. Costs were based on those given by the Rossi model, but modified to reflect the differences in model structures and updated to reflect recent changes in treatment of metastatic kidney cancer and inflation (Table 17). The Rossi model indicates that kidney cancer costs vary by stage at diagnosis and time since diagnosis, with costs in year one being fairly high due to initial treatment, whilst subsequent years only incur costs for a small amount of surveillance unless recurrence occurs [35]. Costs for people with metastatic disease - either diagnosed initially in stage IV or upon progression or recurrence from stage I-III, are extremely large due to the high costs of specialist drugs and palliative care.

The year one costs specified in the Rossi model included the costs of initial assessment and treatment (primarily surgical procedures), and for stages I-III the costs of surveillance across subsequent years were also included in year one costs, with subsequent year costs assumed to be zero. A similar approach was used for this model, but a subset of initial assessment costs referring to a CT scan and blood tests were subtracted from the Rossi costs given that these are costed separately as diagnostic costs in the model (see above). Stage-specific diagnostic assessment costs (primarily for stage IV) were not removed from the Rossi costs as these are not costed separately in the model. Stage I costs were represented by the average of stage Ia and Ib costs as these sub-stages had been defined separately in the Rossi model. Year one costs based on a costing year of 2016 in the Rossi study were inflated to 2022 costs using the NHS Pay and Prices index [30].

Table 17: Unit costs for cancer treatment

| **Health State** | **Unit Cost Rossi** | **Unit Cost Model*** |
| --- | --- | --- |
| Year one stage I | Stage Ia: £7,514; Stage Ib: £8,110  minus £109 initial assessment | £8,700 |
| Year one stage II | £8,743 minus £109 initial assessment | £9,751 |
| Year one stage III | £8,595 minus £109 initial assessment | £9,584 |
| Year one stage IV | £4,555 minus £109 initial assessment | £5,021 |
| Subsequent years all stages | £0 metastasis free costs | £0 |
| Year prior to cancer death, all stages | £759 metastatic recurrence  £1,690 progressive disease  £11,616 terminal care  £1,428 no systemic therapy (28%)  £19,244 1^st^ line therapy (72%)  £47,041 2^nd^ line therapy (47% of 1^st^ line)  £47,041 3rd line therapy (33% of 2^nd^ line) | £136,280  Sum of costs for metastatic recurrence, progressive disease, terminal care and newly costed therapies (see Table 18). |
| *Note includes inflation from 2016 to 2022 | | |

Given that the model does not include a specific progression/recurrence health state, it was assumed that death from cancer would be preceded by progression/recurrence and so those costs were loaded into the year before cancer death, even though in practice people may be in this health state for longer than a year. Due to model structure limitations, this cost was not stage specific, but would be incurred by a higher proportion of the population in late stages than in early stages, thereby creating a cost differential by stage. This cost was assumed to be comprised of the following separate cost categories from the Rossi model added together: metastatic recurrence, progressive disease, terminal care and systemic therapy (Table 17). The first three cost categories were simply inflated, whilst a new costing for systemic therapy was carried out based on the therapies currently recommended by NICE guidelines for metastatic renal cell carcinoma [36-42], with drug costs taken from the British National Formulary [43-51], while monitoring and adverse event costs were inflated from the costs in Rossi’s model. The details of this costing are shown in Table 18.

Table 18: Breakdown of average systemic therapy costs used for metastatic kidney cancer.

| **Terminal Year Costs (all stages)** | | | | | | | | | **Costs** |
| --- | --- | --- | --- | --- | --- | --- | --- | --- | --- |
| Metastatic recurrence | | | | | | | | | £857* |
| Progressive disease | | | | | | | | | £636* |
| Terminal care | | | | | | | | | £13,119* |
| Updated systemic therapy (weighted average all therapy groups) | | | | | | | | | £121,667 |
|  | **Group**  **(% in group)** | **Therapy name(s)**  **(% taking each therapy)** | **Treatment duration (mean months)** | **Treatment dosage (mean daily) [37, 40-42, 52-54]** | **Treatment unit cost per milligram [43-51]** | **Total drug costs** | **Monitoring costs‡** | **Adverse event costs‡** | **Total costs** |
|  | No therapy (28.5%) | NA | NA | NA | NA | NA | NA | NA | £1,075* |
|  | 1st line therapy  (71.5%) | Axitinib + Avelumab (13.3%) | 9 | 10.0mg; 57.1mg | £12.56; £3.84 | £94,454 | £993 | £1,819 | £97,266 |
|  |  | Tivozanib (8.8%) | 17 | 1.0mg | £72.92 | £37,895 | £1,875 | £3,437 | £43,207 |
|  |  | Lenvatinib + Pembrolizumab (54.5%) | 17 | 20.0mg; 9.5mg | £4.79; £26.30 | £179,054 | £1,875 | £3,437 | £184,366 |
|  |  | Ipilimumab + Nivolumab† (23.4%) | 8 | 0.9mg; 15.3mg§ | £75.00; £10.97 | £75,980 | £882 | £1,617 | £78,479 |
|  |  | *Weighted average 1^st^ line therapy costs (100%)* | | | | | | | *£135,590* |
|  | 2nd line therapy  (37.4%) | Cabozantinib (80%) | 8.3 | 60.0mg | £2.86 | £43,280 | £916 | £1,678 | £45,873 |
|  |  | Lenvatinib + Everolimus (20%) | 7.6 | 18.0mg; 5.0mg | £7.98¶; £15.00 | £50,556 | £838 | £1,536 | £52,931 |
|  |  | *Weighted average 2^nd^ line therapy costs (100%)* | | | | | | | *£47,285* |
|  | 3rd line therapy  (14.8%) | Cabozantinib (80%) | 8.3 | 60.0mg | £2.86 | £43,280 | £916 | £1,678 | £45,873 |
|  |  | Axitinib (20%) | 7 | 10.0mg | £12.56 | £26,744 | £772 | £1,415 | £28,931 |
|  |  | *Weighted average 3^rd^ line therapy costs (100%)* | | | | | | | *£34,014* |
|  | 4th line therapy  (5.4%) | Cabozantinib (80%) | 8.3 | 60.0mg | £2.56 | £43,280 | £916 | £1,678 | £45,873 |
|  |  | Axitinib (20%) | 7 | 10.0mg | £12.56 | £26,744 | £772 | £1,415 | £28,931 |
|  |  | *Weighted average 4^th^ line therapy costs (100%)* | | | | | | | *£34,014* |
| **Total terminal year costs** | | | | | | | | | **£136,280** |
| *Costs taken directly from Rossi’s study [35] and inflated to 2022 values. †The dosage depends upon patient weight, which was assumed to be 70kg on average for these calculations. ‡ Costs taken directly from Rossi’s study [35], inflated to 2022 values and scaled to reflect mean treatment duration. § Weighted average of two Nivolumab dosing regimens. ¶Weighted average of two Lenvatinib pack sizes. | | | | | | | | | |

### AAA treatment and surveillance

AAA costs were taken from the English NHS AAA screening programme model [25], inflated to 2022 values using the NHS Pay and Prices cost inflation index [30] (Table 19). The diagnosed small and medium AAA health states were associated with surveillance costs. The frequency of ultrasound surveillance varies depending upon AAA size, being annually for small AAA and four times per year for medium AAA.

Table 19: Costs for AAA treatment and surveillance, taken from the English NHS AAA screening programme model[25].

| **Cost component** | **Unit cost [25]** | **Inflated cost used in model** |
| --- | --- | --- |
| AAA surveillance | £68 | £80 |
| AAA emergency surgery | £19,985 | £23,462 |
| AAA elective surgery | £12,806 | £15,035 |
| Assessment for elective surgery | £435 | £511 |

Different costs were used for emergency and elective surgery, with the elective surgery cohort also incurring an assessment cost. In the absence of other data, it was assumed that costs would be identical whether the patient survived or died following surgery. Costs were incurred only in the first year after surgery.

## Utilities

### Health related quality of life for general population

Health-related quality of life was assumed to vary by age for all population cohorts. A study that calculated EQ-5D by age from Health Survey for England (HSE) data was used to inform quality of life in each cohort at baseline, and as the cohort aged [55]. Data was taken from the general population estimates irrespective of health status, with the value from the age 65-69 group (EQ-5D = 0.8041) being used as the reference standard. Age decrements were assumed to be linear, calculated as 0.00444 for each additional year of age.

Another study based on HSE data indicates that quality of life is lower for smokers compared with non-smokers [56], which is partly due to smoking status and partly due to other correlated characteristics. Data from this study was used in the model to represent the likely reduced baseline quality of life in the modelled population cohorts. Decrements of 0.0169 for ex-smokers and 0.0327 for current smokers (based on the moderate smoker value) were applied across all ages/sexes to the proportion of the population cohort expected to be past/current smokers.

### Utility decrements associated with screening & diagnostic pathways

It was assumed in the basecase analysis that none of the diagnostic procedures were associated with utility decrements. This is incorrect as some procedures have a known risk of harm and this was tested in sensitivity analysis (see section ‎6.2). However, these risks are generally small and may be outweighed by any potential benefits arising from early diagnosis. The model also did not incorporate any long-term health benefits to early detection of incidental findings, so these two omissions work in opposite directions and will cancel out to some extent.

Conceptual modelling identified that it was important to include any harms from screening itself, as this procedure is given to the general population, most of whom will have no disease and derive no benefit from it. There are two potential sources of harm. Firstly there may be anxiety due to screening. A small decrement for anxiety has already been incorporated into evaluation of the targeted lung check [57], so it was thought unlikely that there would be substantial additional anxiety in having two scans at the same time rather than just one. Secondly, there is a small harm caused through the additional radiation that the screening CT produces. Low dose CT as used in this study is expected to result in a very small increased risk of cancer in the future. A one-off utility decrement was estimated per person screened based on the increased cancer risk using the following method:

Utility Decrement = Risk of Cancer per YKST CT Scan * Weighted Average Loss-of-Quality-Adjusted Life Expectancy (QALE) per Cancer Case

The atomic bomb study indicates that the risk of all solid cancers is consistent with a linear increase in radiation dose [58]. The risk of fatal malignancy from radiation exposure was estimated at 0.00005 per unit effective radiation dose (mSv) for an abdominal CT scan [59]. It was assumed that the risk of cancer from radiation exposure in a smoking population was the same as in the general population. In the YKST final iteration, the average effective dose of one CT scan was 2.01 mSv. Combining these two values, the expected lifetime risk of cancer from one YKST scan was estimated at 0.0001.

QALE loss data for selected cancers was extracted from a study in Taiwan [60] as there were no equivalent data sources found from the UK or more appropriate countries. The weighted average loss-of-QALE was calculated across multiple abdominal cancers (kidney, liver, bowel, stomach, bladder, renal pelvis and ureter) based on cancer proportions from NDRS 2019 [11]. Values given for each sex were weighted equally. The weighted loss-of-QALE was estimated at 7.77 per cancer case.

Combining these two values resulted in an estimated utility decrement of 0.00078 per person screened in YKST. This was applied as a one-off decrement to the population in the screening arm of the model.

### Utility decrements associated with cancer

A cancer diagnosis results in a reduction in utility primarily due to the impact of treatment. Whilst in practice some people will suffer symptoms prior to diagnosis, this will only happen transiently prior to diagnosis in the non-screening arm of the model, so it was assumed for simplicity that there would be no reduction in health related quality of life for undiagnosed cancer health states.

Recently, quality of life data has been routinely collected 18 months post-diagnosis of cancer from people in the UK through the Cancer Quality of Life Survey [61]. The EQ-5D index summary score data was extracted for use. Data for all cancers combined shows a clear stage distribution with more severe stages having lower quality of life; however the data for individual cancers does not clearly indicate this in all cases due to small numbers. In order to accurately represent the expected stage differences, cancer-type specific data for all stages was combined with stage-specific data for all cancers to create a set of stage-specific values for each cancer type. Data for both kidney cancer and ureteric cancer were derived from the kidney plus urinary tract cancer values. There was a lack of data for the rarer cancer types so it was assumed that data for gallbladder cancer was the same as liver cancer, adrenal cancer was the same as kidney cancer and Hodgkin lymphoma was the same as non-Hodgkin lymphoma.

The stage and cancer-type specific data, which represent absolute quality of life values, were then converted to a set multipliers based on the expected quality of life in someone from the general population aged 66 estimated based on EQ-5D in HSE data [55] (the average age of cancer diagnosis is 66.4 according to the UK Biobank [62]. This enabled the multipliers to be used together with the age-specific baseline quality of life assigned to each population cohort.

The final step was to reflect differences in quality of life by time since diagnosis. Quality of life is generally poorer in the first year after diagnosis due to surgery and other treatments undergone, and then improves subsequently. However, there is some evidence that it remains poorer on average in cancer patients after remission than in the general population for many years post diagnosis [63]. Quality of life also reduces after progression/recurrence.

A rapid review of published articles indicated that there was little consistent data around EQ-5D changes over time across cancer types; however, a study was identified that measured quality of life over time for multiple cancers, based on the short-form 6-dimension (SF-6D) questionnaire [63]. This indicated that compared to someone without cancer, there was an average decrement of 0.048 in people in remission <2 years post diagnosis, 0.056 in people who were not in remission and 0.014-0.02 in people in remission between >2 and >10 years after diagnosis. It was assumed the UK Cancer Quality of life data represented people in remission <2 years, whilst utilities in year one and the year prior to cancer death (assumed to represent progression/recurrence) were assumed to be equivalent to the ‘not in remission’ data). Utilities in subsequent years were assumed be equivalent to the average data from people in remission between >2 and >10 years. Multipliers were calculated using these values in order to reduce or increase the cancer/stage-specific values to represent the utilities in the year one or subsequent year diagnosed health states (Table 20).

Table 20: Estimated utility multipliers for cancer by cancer type, stage and time since diagnosis, based on Cancer Quality of Life survey data [61].

| **Cancer Type** | **Time Since Diagnosis** | **Stage I** | **Stage II** | **Stage III** | **Stage IV** |
| --- | --- | --- | --- | --- | --- |
| Kidney | Year one/progression | 0.845 | 0.793 | 0.791 | 0.683 |
|  | Subsequent years | 0.950 | 0.934 | 0.933 | 0.898 |
| Liver | Year one/progression | 0.797 | 0.747 | 0.745 | 0.640 |
|  | Subsequent years | 0.935 | 0.919 | 0.918 | 0.884 |
| Stomach | Year one/progression | 0.804 | 0.754 | 0.752 | 0.646 |
|  | Subsequent years | 0.937 | 0.921 | 0.920 | 0.886 |
| Oesophageal | Year one/progression | 0.830 | 0.779 | 0.777 | 0.670 |
|  | Subsequent years | 0.946 | 0.929 | 0.928 | 0.894 |
| Pancreatic | Year one/progression | 0.799 | 0.749 | 0.747 | 0.642 |
|  | Subsequent years | 0.936 | 0.919 | 0.919 | 0.885 |
| Colon | Year one/progression | 0.938 | 0.884 | 0.881 | 0.768 |
|  | Subsequent years | 0.980 | 0.963 | 0.962 | 0.925 |
| Ureteric | Year one/progression | 0.845 | 0.793 | 0.791 | 0.683 |
|  | Subsequent years | 0.950 | 0.934 | 0.933 | 0.898 |
| Gallbladder | Year one/progression | 0.797 | 0.747 | 0.745 | 0.640 |
|  | Subsequent years | 0.935 | 0.919 | 0.918 | 0.884 |
| Adrenal | Year one/progression | 0.845 | 0.793 | 0.791 | 0.683 |
|  | Subsequent years | 0.950 | 0.934 | 0.933 | 0.898 |
| Hodgkin Lymphoma | Year one/progression | 0.883 | 0.830 | 0.828 | 0.718 |
|  | Subsequent years | 0.962 | 0.945 | 0.945 | 0.909 |
| Non-Hodgkin Lymphoma | Year one/progression | 0.883 | 0.830 | 0.828 | 0.718 |
|  | Subsequent years | 0.962 | 0.945 | 0.945 | 0.909 |

### Utility decrements associated with AAA

Whilst it seems plausible that utility decrements could be associated with anxiety around having diagnosed AAA that is too small to operate on, or in people who are recovering from surgery or who suffer surgical complications, no utility decrements due to AAA have been used in previous AAA modelling of the English population [25, 26]. In order to make results comparable against the existing AAA modelling, no AAA utility decrements were therefore included in the model.

# Model analyses

## Primary model analyses

The basecase model analyses compared UA screening against the comparator (no UA screening) in an incremental analysis. Probabilistic sensitivity analysis (PSA) was implemented for all analyses in order to obtain accurate estimates of mean outcomes in the non-linear model, and to investigate the impact of uncertainty around parameter values. For each analysis, 2000 PSA samples were run and results averaged. The basecase analyses included results for the following population compositions:

- A population distribution representing the eligible population for the lung health checks in England. This was composed of cohorts aged 55-74, with each age and sex group weighted to reflect the proportions in the YKST study.
- Results for each age/sex group separately, in order to assess the optimal age for a one-off screen.

## Scenario analyses

In addition, a set of scenario analyses were carried out in order to investigate the impact of structural uncertainty (also using PSA). The following analyses were performed:

1. **Cancer stage specific mortality after screening.** There is some evidence from bowel cancer screening that stage-specific survival could be better in people diagnosed through screening than those diagnosed symptomatically [24]. However, other evidence indicates that screening does not always produce mortality benefits despite stage shift [64]. A sensitivity analysis was carried out in which stage-specific mortality is reduced in the screening arm uniformly across all cancer types, stages, ages, sexes and at all time periods after diagnosis, by a proportion equivalent to that found in bowel cancer screening[24]. This sensitivity analysis is likely to overestimate the benefits of screening as some of the mortality benefits seen with bowel cancer screening can be explained by other factors such as differences between populations who take up screening and those who don’t, and it also may not be transferable to other cancers. A second sensitivity analysis has also been done where stage specific mortality is reduced in the screening arm by half the amount observed for bowel cancer.
2. **Cancer stage distribution at screening.** The benefits of screening are driven through the differences in stage distribution in screening and comparator arms. However, whilst stage distribution in comparator arms is based on robust national data, there is significant uncertainty around the calculated stage distribution at screening, due to the lack of empirical data to inform this, which goes beyond the incorporated parameter uncertainty. Furthermore, given that CT sensitivity will in general be higher for larger cancers, this will inhibit the ability of screening to find as many early cancers, thereby reducing stage shift impacts. To investigate the impact of a much more conservative estimate of benefit, a sensitivity analysis was carried out in which the stage shift was assumed to be half that of the calculated values. In addition, smaller impacts on stage shift were also tested to examine the impact of either a 25% increase or decrease in the proportion of stage 4 cancers at screening compared to the basecase.
3. **Cancer treatment costs.** The cost of metastatic cancer treatment implemented in the model is highly uncertain as the treatments are subject to confidential patient access schemes, which are likely to considerably reduce their costs. In addition, it was assumed that all cancer treatments would cost the same as kidney cancer treatment, whereas in fact this is unlikely to be true, meaning that there is additional uncertainty around all treatment costs for other cancer types. To test the sensitivity of the model to treatment costs, several sensitivity analyses were carried out where a) metastatic treatment costs were halved for all cancers; b) metastatic treatment costs were halved for all cancers apart from kidney cancer; c) metastatic treatment costs were doubled for all cancers; d) metastatic treatment costs were doubled for all cancers apart from kidney cancer; e) all cancer treatment costs were halved.
4. **Cancer prevalence at screening.** It may be possible to select people at higher risk for kidney or other cancers than the current eligible population, which would enrich the number of cancers found through screening. Equally, given uncertainty around the prevalence estimates given the low number of cases found in YKST, it is possible that the basecase scenario could overestimate cancer prevalence. Sensitivity analyses were carried out where risk (incorporated in the model as underlying prevalence) for either kidney cancer or all cancers was either doubled or halved.
5. **Utility decrements in people with secondary findings**. The basecase analysis only includes the impact of secondary findings in terms of their diagnostic costs, based on YKST data. An additional analysis was carried out whereby all secondary findings also incur a utility decrement related to the additional diagnostic procedures required. This was based on the decrement used for having a kidney biopsy (-0.006) from a modelling study for small renal mass management [65]. Note that this is likely to overestimate the harm of secondary findings as most people had non-serious findings that did not require invasive follow-up, and it ignores any long-term benefits of early diagnosis.
6. **Population age distribution**. The basecase analysis only includes the age range of people included in the lung health checks. However, YKST enrolled some older people. An additional analysis was carried out in which the full YKST population (age 55-81) was modelled.
7. **Discount rates.** These were a) reduced to 1.5%; b) increased to 5% in sensitivity analysis.
8. **Assessing the impact of removing some diseases from the findings.** The model used for this set of analyses is unusual in its inclusion of multiple conditions. A set of analyses were carried out to test the impact of modelling upper abdominal screening without including the full range of modelled conditions. This included a) removing AAA findings; b) removing all findings other than AAA; c) removing all findings apart from kidney cancer and secondary kidney findings.
9. **Screening costs.** Screening costs implemented in the primary analysis reflect expected resource use if the intervention were to be rolled out in practice. A maximum justifiable cost analysis was carried out to assess what the maximum costs of screening could be whilst enabling the intervention to remain cost-effective.

## Value of information analyses

Value of information analyses were undertaken using the Sheffield Accelerated Value of Information tool (SAVI) [66] to determine the value of reducing uncertainty in the model results. This used the PSA results from the primary analysis to estimate the total expected value of having perfect information (EVPI) based on the joint parameter uncertainty. In addition, expected value of partially perfect information (EVPPI) was performed to investigate which parameters and parameter groups were contributing most significantly to the uncertainty, which will help inform the design of a full trial.

# References

1. Cumberbatch, M.G., et al., *The Role of Tobacco Smoke in Bladder and Kidney Carcinogenesis: A Comparison of Exposures and Meta-analysis of Incidence and Mortality Risks.* Eur Urol, 2016. **70**(3): p. 458-66.

2. Lee, Y.C., et al., *Meta-analysis of epidemiologic studies on cigarette smoking and liver cancer.* Int J Epidemiol, 2009. **38**(6): p. 1497-511.

3. Ladeiras-Lopes, R., et al., *Smoking and gastric cancer: systematic review and meta-analysis of cohort studies.* Cancer Causes Control, 2008. **19**(7): p. 689-701.

4. Tramacere, I., C. La Vecchia, and E. Negri, *Tobacco smoking and esophageal and gastric cardia adenocarcinoma: a meta-analysis.* Epidemiology, 2011. **22**(3): p. 344-9.

5. Bosetti, C., et al., *Cigarette smoking and pancreatic cancer: an analysis from the International Pancreatic Cancer Case-Control Consortium (Panc4).* Ann Oncol, 2012. **23**(7): p. 1880-8.

6. Cheng, J., et al., *Meta-analysis of prospective cohort studies of cigarette smoking and the incidence of colon and rectal cancers.* Eur J Cancer Prev, 2015. **24**(1): p. 6-15.

7. Van Osch, F.H., et al., *Quantified relations between exposure to tobacco smoking and bladder cancer risk: a meta-analysis of 89 observational studies.* International Journal of Epidemiology, 2016. **45**(3): p. 857-870.

8. McGee, E.E., et al., *Smoking, Alcohol, and Biliary Tract Cancer Risk: A Pooling Project of 26 Prospective Studies.* J Natl Cancer Inst, 2019. **111**(12): p. 1263-1278.

9. Yousaf, A., et al., *Smoking is associated with adrenal adenomas and adrenocortical carcinomas: a nationwide multicenter analysis.* Cancer Treat Res Commun, 2020. **25**: p. 100206.

10. Sergentanis, T.N., et al., *Cigarette smoking and risk of lymphoma in adults: a comprehensive meta-analysis on Hodgkin and non-Hodgkin disease.* Eur J Cancer Prev, 2013. **22**(2): p. 131-50.

11. *CancerData: Cancer Incidence and Mortality*. 2019 [cited 2024 15th February]; Available from: <https://www.cancerdata.nhs.uk/incidence_and_mortality>.

12. Brown, K.F., et al., *The fraction of cancer attributable to modifiable risk factors in England, Wales, Scotland, Northern Ireland, and the United Kingdom in 2015.* Br J Cancer, 2018. **118**(8): p. 1130-1141.

13. *Adult smoking habits in England*. 2021 [cited 2024 15th February]; Available from: <https://www.ons.gov.uk/peoplepopulationandcommunity/healthandsocialcare/healthandlifeexpectancies/datasets/adultsmokinghabitsinengland>.

14. Schwartzberg, L., et al., *Impact of early detection on cancer curability: A modified Delphi panel study.* PLoS One, 2022. **17**(12): p. e0279227.

15. Broder, M.S., et al., *Estimates of stage-specific preclinical sojourn time across 21 cancer types.* Journal of Clinical Oncology, 2021. **39**(15_suppl): p. e18584-e18584.

16. *Staging data in England*. 2019 [cited 2024 15th February]; Available from: <https://nhsd-ndrs.shinyapps.io/staging_data_in_england/>.

17. *Cancer survival in England - adults diagnosed*. 2017 [cited 2024 15th February]; Available from: <https://www.ons.gov.uk/peoplepopulationandcommunity/healthandsocialcare/conditionsanddiseases/datasets/cancersurvivalratescancersurvivalinenglandadultsdiagnosed>.

18. Henley, S.J., et al., *Gallbladder Cancer Incidence and Mortality, United States 1999-2011.* Cancer Epidemiol Biomarkers Prev, 2015. **24**(9): p. 1319-26.

19. Sharma, E., et al., *The Characteristics and Trends in Adrenocortical Carcinoma: A United States Population Based Study.* Journal of Clinical Medicine Research, 2018. **10**(8): p. 636-640.

20. Robinson, A.G., et al., *Is cancer stage data missing completely at random? A report from a large population-based cohort of non-small cell lung cancer.* Frontiers in Oncology, 2023. **13**.

21. Rossi, S.H., et al., *Meta-analysis of the prevalence of renal cancer detected by abdominal ultrasonography.* Br J Surg, 2017. **104**(6): p. 648-659.

22. Menon, U., et al., *Ovarian cancer population screening and mortality after long-term follow-up in the UK Collaborative Trial of Ovarian Cancer Screening (UKCTOCS): a randomised controlled trial.* The Lancet, 2021. **397**(10290): p. 2182-2193.

23. Martin, R.M., et al., *Effect of a Low-Intensity PSA-Based Screening Intervention on Prostate Cancer Mortality.* JAMA, 2018. **319**(9): p. 883.

24. Cardoso, R., et al., *Overall and stage-specific survival of patients with screen-detected colorectal cancer in European countries: A population-based study in 9 countries.* The Lancet Regional Health - Europe, 2022. **21**: p. 100458.

25. Glover, M.J., et al., *Cost-effectiveness of the National Health Service abdominal aortic aneurysm screening programme in England.* British Journal of Surgery, 2014. **101**(8): p. 976-982.

26. Thompson, S.G., et al., *Screening women aged 65 years or over for abdominal aortic aneurysm: a modelling study and health economic evaluation.* Health Technology Assessment, 2018. **22**(43): p. 1-142.

27. *National life tables: England*. 2020 [cited 2024 1st March]; Available from: <https://www.ons.gov.uk/peoplepopulationandcommunity/birthsdeathsandmarriages/lifeexpectancies/datasets/nationallifetablesenglandreferencetables>.

28. *Deaths registered summary statistics, England and Wales*. 2021 [cited 2024 1st March]; Available from: <https://www.ons.gov.uk/peoplepopulationandcommunity/birthsdeathsandmarriages/deaths/datasets/deathsregisteredsummarystatisticsenglandandwales>.

29. Pirie, K., et al., *The 21st century hazards of smoking and benefits of stopping: a prospective study of one million women in the UK.* Lancet, 2013. **381**(9861): p. 133-41.

30. Jones, K., et al., *Unit Costs of Health and Social Care 2022*. 2022: Personal Social Services Research Unit, University of Kent, Canterbury.

31. *NICE health technology evaluations: the manual*. 2022 [cited 2024 1st March]; Available from: <https://www.nice.org.uk/process/pmg36/chapter/introduction-to-health-technology-evaluation>.

32. Lyratzopoulos, G., et al., *Measures of promptness of cancer diagnosis in primary care: secondary analysis of national audit data on patients with 18 common and rarer cancers.* Br J Cancer, 2013. **108**(3): p. 686-90.

33. *Emergency presentations of cancer*. [cited 2024 1st March]; Available from: <https://www.cancerdata.nhs.uk/emergencypresentations>.

34. *National Cost Collection for the NHS*. 2022 [cited 2024 1st March]; Available from: <https://www.england.nhs.uk/costing-in-the-nhs/national-cost-collection/>.

35. Rossi, S.H., et al., *A Decision Analysis Evaluating Screening for Kidney Cancer Using Focused Renal Ultrasound.* Eur Urol Focus, 2021. **7**(2): p. 407-419.

36. *Avelumab with axitinib for untreated advanced renal cell carcinoma, Technology appraisal guidance, TA645*. 2020 [cited 2024 4th March]; Available from: <https://www.nice.org.uk/guidance/ta645>.

37. *Tivozanib for treating advanced renal cell carcinoma, Technology appraisal guidance, TA512*. 2018 [cited 2024 4th March]; Available from: <https://www.nice.org.uk/guidance/ta512>.

38. *Lenvatinib with pembrolizumab for untreated advanced renal cell carcinoma, Technology appraisal guidance, TA858*. 2023 [cited 2024 4th March]; Available from: <https://www.nice.org.uk/guidance/ta858>.

39. *Nivolumab with ipilimumab for untreated advanced renal cell carcinoma, Technology appraisal guidance, TA780*. 2022 [cited 2024 4th March]; Available from: <https://www.nice.org.uk/guidance/ta780>.

40. *Cabozantinib for previously treated advanced renal cell carcinoma, Technology appraisal guidance, TA463*. 2017 [cited 2024 4th March]; Available from: <https://www.nice.org.uk/guidance/ta463>.

41. *Lenvatinib with everolimus for previously treated advanced renal cell carcinoma, Technology appraisal guidance, TA498*. 2018 [cited 2024 4th March]; Available from: <https://www.nice.org.uk/guidance/ta498>.

42. *Axitinib for treating advanced renal cell carcinoma after failure of prior systemic treatment, Technology appraisal guidance, TA333*. 2015 [cited 2024 4th March]; Available from: <https://www.nice.org.uk/guidance/ta333>.

43. *Axitinib [Specialist drug] Medicinal forms, British National Formulary (BNF)*. 2023 [cited 2024 6th March]; Available from: <https://bnf.nice.org.uk/drugs/axitinib-specialist-drug/medicinal-forms/>.

44. *Avelumab [Specialist drug] Medicinal forms, British National Formulary (BNF)*. 2023 [cited 2024 6th March]; Available from: <https://bnf.nice.org.uk/drugs/avelumab-specialist-drug/medicinal-forms/>.

45. *Tivozanib [Specialist drug] Medicinal forms, British National Formulary (BNF)*. 2023 [cited 2024 6th March]; Available from: <https://bnf.nice.org.uk/drugs/tivozanib-specialist-drug/medicinal-forms/>.

46. *Lenvatinib [Specialist drug] Medicinal forms, British National Formulary (BNF)*. 2023 [cited 2024 6th March]; Available from: <https://bnf.nice.org.uk/drugs/lenvatinib-specialist-drug/medicinal-forms/>.

47. *Pembrolizumab [Specialist drug] Medicinal forms, British National Formulary (BNF)*. 2023 [cited 2024 6th March]; Available from: <https://bnf.nice.org.uk/drugs/pembrolizumab-specialist-drug/medicinal-forms/>.

48. *Ipilimumab [Specialist drug] Medicinal forms, British National Formulary (BNF)*. 2023 [cited 2024 6th March]; Available from: <https://bnf.nice.org.uk/drugs/ipilimumab-specialist-drug/medicinal-forms/>.

49. *Nivolumab [Specialist drug] Medicinal forms, British National Formulary (BNF)*. 2023 [cited 2024 6th March]; Available from: <https://bnf.nice.org.uk/drugs/nivolumab-specialist-drug/medicinal-forms/>.

50. *Cabozantinib [Specialist drug] Medicinal forms, British National Formulary (BNF)*. 2023 [cited 2024 6th March]; Available from: <https://bnf.nice.org.uk/drugs/cabozantinib-specialist-drug/medicinal-forms/>.

51. *Everolimus Medicinal forms, British National Formulary (BNF)*. 2023 [cited 2024 6th March]; Available from: <https://bnf.nice.org.uk/drugs/everolimus/medicinal-forms/>.

52. *Bavencio 20 mg/mL concentrate for solution for infusion, summary of product characteristics for avelumab, electronic medicines compendium (emc)*. 2023 [cited 2024 6th March]; Available from: <https://www.medicines.org.uk/emc/product/8453/smpc>.

53. *Kisplyx 10 mg hard capsules, summary of product characteristics for lenvatinib, electronic medicines compendium (emc)*. 2023 [cited 2024 6th March]; Available from: <https://www.medicines.org.uk/emc/product/7881/smpc>.

54. *OPDIVO 10 mg/mL concentrate for solution for infusion, summary of product characteristics for nivolumab, electronic medicines compendium (emc)*. 2023 [cited 2024 6th March]; Available from: <https://www.medicines.org.uk/emc/product/6888/smpc>.

55. Ara, R. and J.E. Brazier, *Using Health State Utility Values from the General Population to Approximate Baselines in Decision Analytic Models when Condition-Specific Data are Not Available.* Value in Health, 2011. **14**(4): p. 539-545.

56. Vogl, M., et al., *Smoking and health-related quality of life in English general population: implications for economic evaluations.* BMC Public Health, 2012. **12**: p. 203.

57. Snowsill, T., et al., *Low-dose computed tomography for lung cancer screening in high-risk populations: a systematic review and economic evaluation.* Health Technology Assessment, 2018. **22**(69): p. 1-276.

58. Hall, E.J. and D.J. Brenner, *Cancer risks from diagnostic radiology.* The British Journal of Radiology, 2008. **81**(965): p. 362-378.

59. Gerber, T.C., et al., *Ionizing Radiation in Cardiac Imaging.* Circulation, 2009. **119**(7): p. 1056-1065.

60. Lai, W.-W., et al., *QALYs and medical costs saved from prevention of a cancer: Analysis of nation-wide real-world data of Taiwan with lifetime horizon.* Journal of the Formosan Medical Association, 2021. **120**(12): p. 2089-2099.

61. *Cancer quality of life survey*. [cited 2024 1st March]; Available from: <https://digital.nhs.uk/ndrs/data/data-outputs/cancer-data-hub/cancer-quality-of-life-survey>.

62. *UK Biobank Malignant Cancer Summary Report*. 2023: UK Biobank.

63. Wang, S.-Y., et al., *Association between Time since Cancer Diagnosis and Health-Related Quality of Life: A Population-Level Analysis.* Value in Health, 2016. **19**(5): p. 631-638.

64. Chen, X., et al., *Prognostic Significance of Blood-Based Multi-cancer Detection in Plasma Cell-Free DNA.* Clinical Cancer Research, 2021. **27**(15): p. 4221-4229.

65. McAlpine, K., et al., *Optimizing the management of patients with small renal masses in a Canadian context: A Markov decision-analysis model.* Can Urol Assoc J, 2022. **16**(1): p. E32-E38.

66. Strong, M., J.E. Oakley, and A. Brennan, *Estimating multiparameter partial expected value of perfect information from a probabilistic sensitivity analysis sample: a nonparametric regression approach.* Med Decis Making, 2014. **34**(3): p. 311-26.
