# Supplementary material for: Cost-effectiveness of one-off upper abdominal CT screening as an add-on to lung cancer screening in England
Source: Br J Cancer. 2025 May 14;133(2):239–47. doi: 10.1038/s41416-025-03043-z (PMC12304199; doi:10.1038/s41416-025-03043-z)

**Supplementary Tables and Figures**

**Cost-effectiveness of upper abdominal CT screening for kidney cancer as an add-on to lung cancer screening in England**

Table S1: Comparison of estimated number of disease cases for the YKST population versus actual number of upper-abdominal scan-specific disease cases observed in YKST (YKST N = 4019).

| **Cancer Type** | **Model estimated (95% CI)** | **YKST observed (95% CI)** |
| --- | --- | --- |
| Kidney | 9.95 (4.56-15.57) | 10 (4.8-17.1) |
| Liver | 2.70 (0.99-4.54) | 2 (0.24-5.6) |
| Stomach | 0.93 (0.42-1.58) | 0 |
| Oesophageal* | 0 | 1 (0.025-3.7) |
| Pancreatic | 3.10 (1.2-4.97) | 1 (0.025-3.7) |
| Colon | 2.29 (0.12-4.97) | 1 (0.025-3.7) |
| Upper urinary tract | 3.36 (1.94-5.37) | 2 (0.24-5.6) |
| Gallbladder | 0.26 (0.09-0.4) | 0 |
| Adrenal | 0.11 (0.05-0.17) | 0 |
| Lymphoma | 1.77 (0.84-3.03) | 2 (0.24-5.6) |
| AAA | 60.47 (50.25-72.51) | 60 (45.87-75.96) |
| YKST Yorkshire Kidney Screening Trial; 95% CI = credible intervals for model results; 95% CI = confidence intervals for YKST observed results; NA not applicable; *Although one oesophageal cancer was found in YKST, the clinical team advised that in practice this would have been found in the lung scan. | | |

Table S2: Disease cases and deaths expected in each modelled arm and incrementally, in the basecase analysis for the population aged 55-74. Results are per 100,000 people scanned, over the remaining lifetime of the screening population. Note that total numbers of cases and deaths given for each model arm refer specifically to those who would be detected through upper abdominal screening, and do not include pre-existing known disease, disease detected through the lung scan, disease missed by upper abdominal screening or disease developed later.

| **Disease** | **Disease Cases per 100,000 Screened** | | | **Disease Deaths per 100,000 Screened** | | |
| --- | --- | --- | --- | --- | --- | --- |
|  | Current Care | Screening | **Incremental** | Current Care | Screening | **Incremental** |
| Kidney Cancer | 206 | 225 | **19.5** | 126 | 108 | **-17.6** |
| Liver Cancer | 54 | 57 | **3.4** | 49 | 49 | **0.3** |
| Stomach Cancer | 17 | 18 | **1.8** | 15 | 14 | **-1.1** |
| Pancreatic Cancer | 61 | 64 | **3.3** | 58 | 60 | **2.1** |
| Colon Cancer | 43 | 48 | **4.7** | 25 | 15 | **-10.6** |
| Upper Urinary Tract Cancer | 61 | 68 | **6.5** | 39 | 36 | **-2.9** |
| Gallbladder Cancer | 5 | 5 | **0.3** | 4 | 4 | **0.04** |
| Adrenal Cancer | 2 | 3 | **0.3** | 1 | 1 | **-0.3** |
| Lymphomas | 35 | 39 | **4.4** | 23 | 21 | **-1.2** |
| AAA | NA* | 1233 | **NA*** | 230 | 88 | **-142** |
| AAA Abdominal aortic aneurysm; NA Not available; *Model structure means current care AAA diagnoses not collected | | | | | | |

Table S3: Resource use incurred and averted by screening, per 100,000 people scanned, in the basecase analysis for the population aged 55-74. Note that resource use incurred only includes additional up-front resource incurred as a direct consequence of screening, based on data taken directly from YKST, and does not take into account any later reduction in resources that might happen due to early diagnosis, or increase in resources that might be needed to manage diagnosed people. GP appointments and emergency presentations averted relate specifically to later diagnosis of cancer.

| **Resource** | **Up-Front Use Incurred by Screening** |
| --- | --- |
| Screening procedures (see Table 1) | 100,000 |
| Blood tests | 7,281 |
| CT scans | 4,742 |
| Urine tests | 391 |
| Ultrasound | 373 |
| Other kidney procedures | 351 |
| MRI | 326 |
| Biopsy: abdomen | 187 |
| Biopsy: kidney | 95 |
| ERCP | 91 |
| Colonoscopy/endoscopy | 64 |
| Biopsy: liver | 62 |
| **Resource** | **Use Averted by Screening** |
| GP visits (symptomatic cancer) | 840 |
| Emergency presentations (symptomatic cancer) | 124 |
| GP General Practitioner; CT computer tomography; MRI magnetic resonance imaging; ERCP endoscopic retrograde cholangiopancreatogram; ECG electrocardiogram | |

Table S4: Expected value of partially perfect information (EVPPI) results showing the contribution of different parameter types to the value of total parameter uncertainty. EVPPI is shown per person scanned. Note that EVPPI for each parameter/parameter group does not and is not expected to sum to total EVPI. Some parameter groups were too large to combine (e.g. cancer incidence and survival) so values reported relate to separate cancer types.

| **Parameter set** | **Per person EVPPI (£)** |
| --- | --- |
| Total EVPI (All parameters) | 0.379 |
| Cancer dwell times | 0.052 |
| AAA prevalence & Markov transitions | 0.027 |
| Cancer survival rates (for separate cancer types) | 0 to 0.023 |
| All model utilities combined | 0.013 |
| Cancer incidence rates (for separate cancer types) | 0 to 0.007 |
| Screening sensitivity estimates | 0.006 |
| All model resource use combined | 0.006 |
| All adjustments made for modelling smokers | 0.004 |
| Current care stage distribution | 0.004 |
| All model costs combined | 0.001 |
| AAA Abdominal aortic aneurysm; EVPI Expected value of perfect information; EVPPI Expected value of partially perfect information | |

Figure S5: Proportion of diagnosed cancers distributed in each stage (I to IV) in current care (black, based on current stage distribution data) and expected stage distribution as detected through upper-abdominal screening (yellow, based on model calculations as described in the supplementary methods file), for the cancer types included in the model.


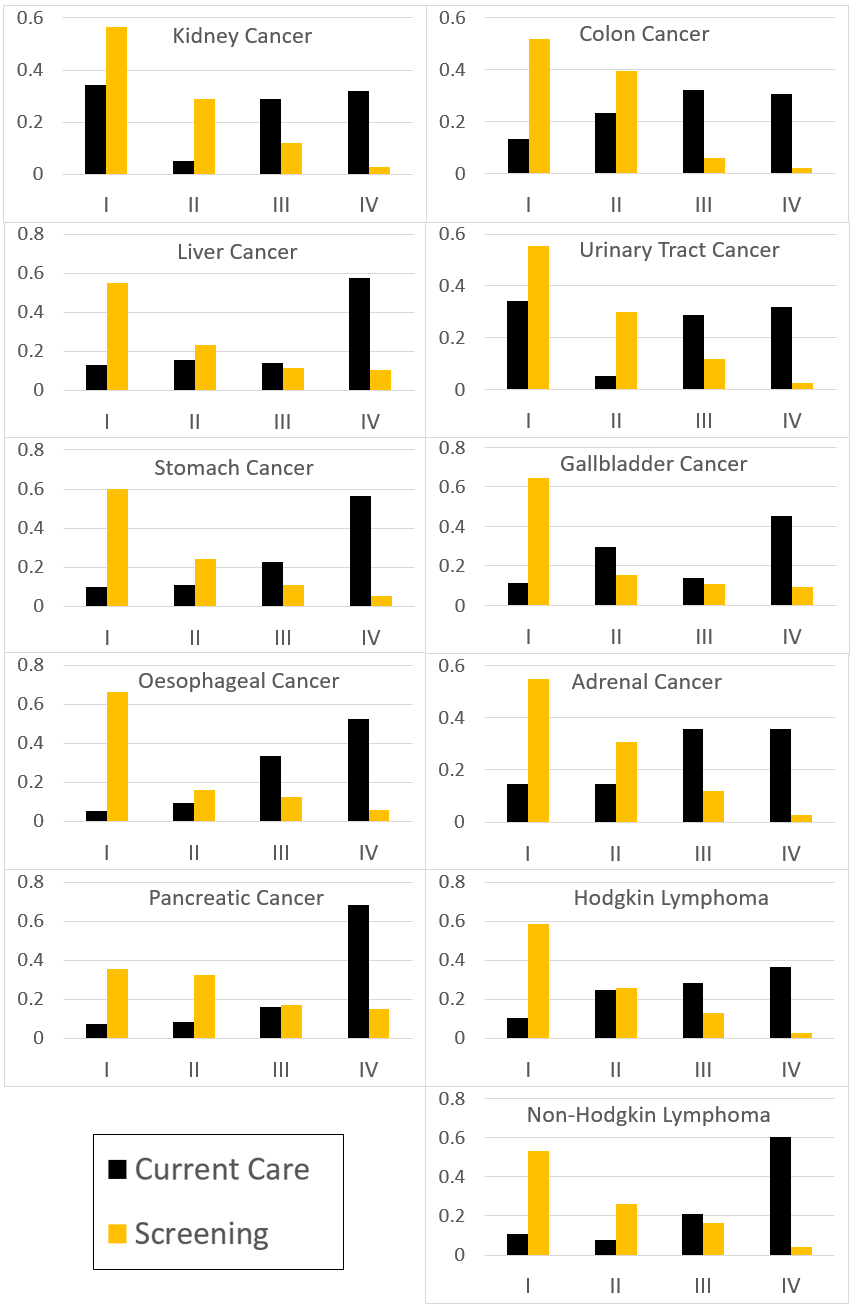


Figure S6: Cost-effectiveness results comparing upper abdominal screening as an add-on to lung screening, to lung screening only (current care) for separate age and sex cohorts; Left: Distribution of probabilistic results (incremental per person costs and QALYs) on the cost-effectiveness plane. The red point indicates the probabilistic mean and the dotted line represents the £20,000 per QALY threshold; Right: Cost-effectiveness acceptability curves showing the probability that upper abdominal screening is cost-effective at different willingness-to-pay thresholds.


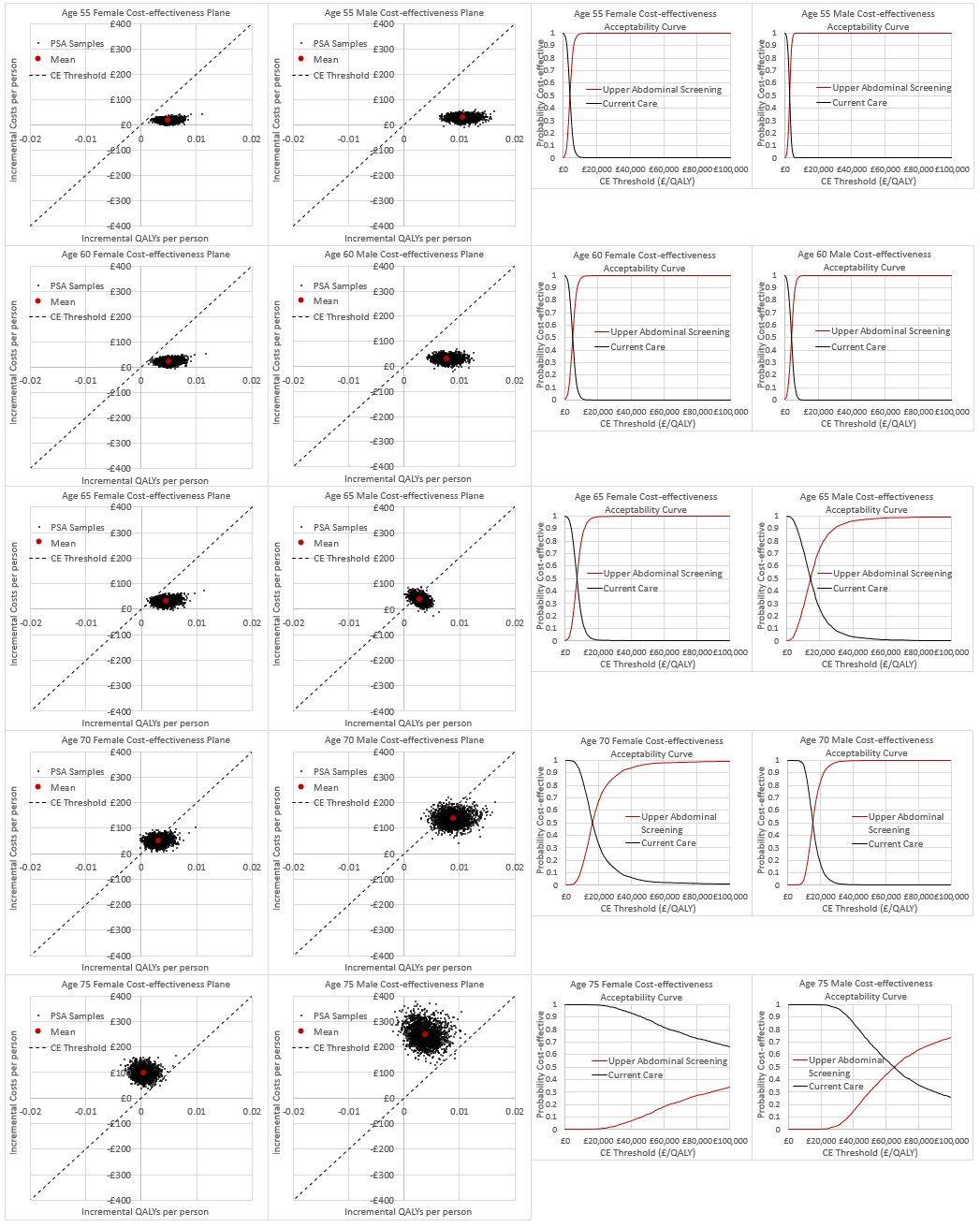


Figure S7: Scenario analysis results: Cost-effectiveness results comparing upper abdominal screening as an add-on to lung screening, to lung screening only (current care) under different structural assumptions; Right: Cost-effectiveness acceptability curves showing the probability that upper abdominal screening is cost-effective at different willingness-to-pay thresholds; Left: Distribution of probabilistic results (incremental per person costs and QALYs) on the cost-effectiveness plane. The red point indicates the probabilistic mean and the dotted line represents the £20,000 per QALY threshold.


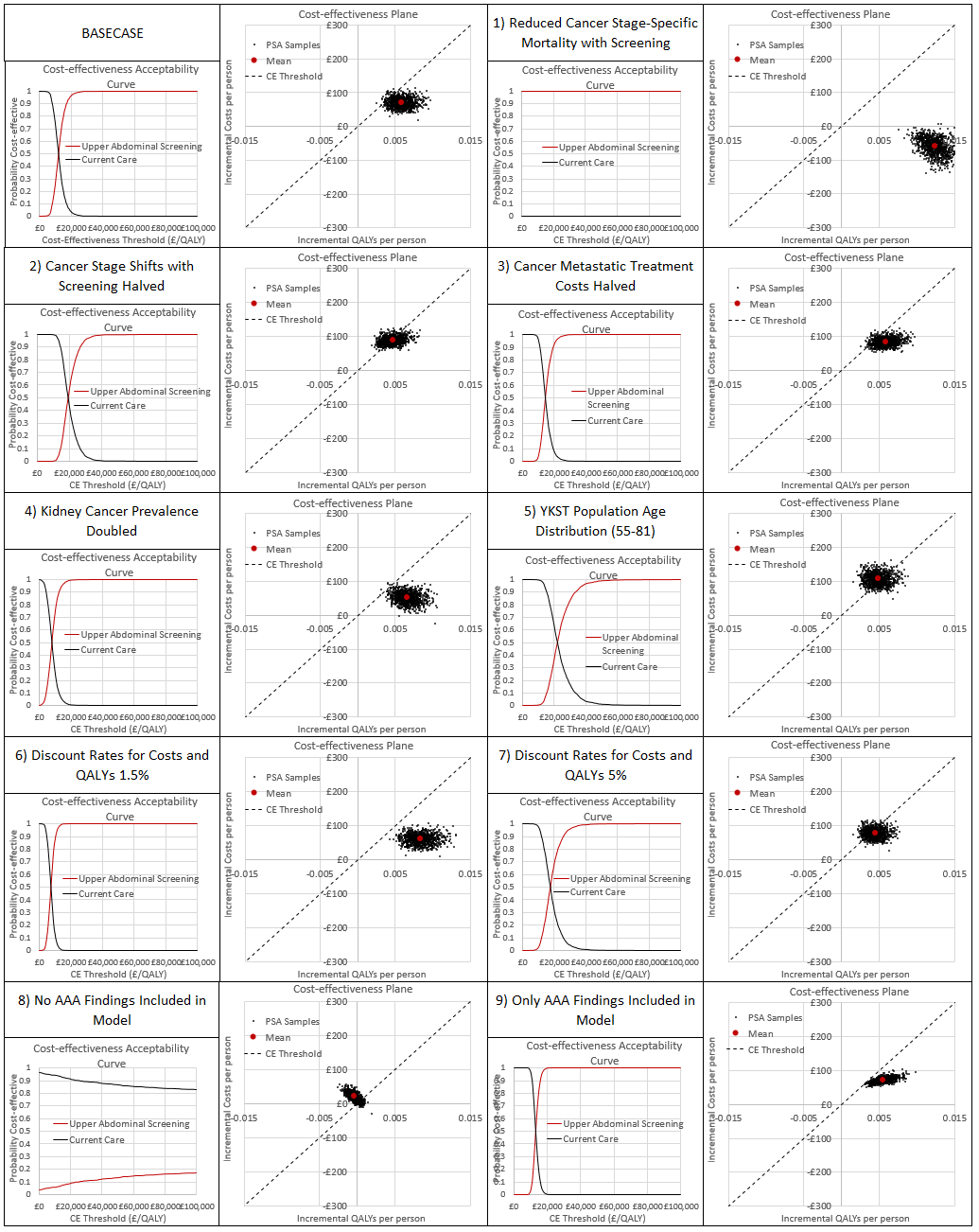


Figure S8: Structural scenario analyses either including only kidney findings in the model results or excluding AAA findings specifically from the model results. Graphs showing how per person incremental cost-effectiveness results change by age and sex of the screening population for incremental net monetary benefit (based on a willingness-to-pay threshold of £20,000 per QALY), incremental QALYs and incremental costs.


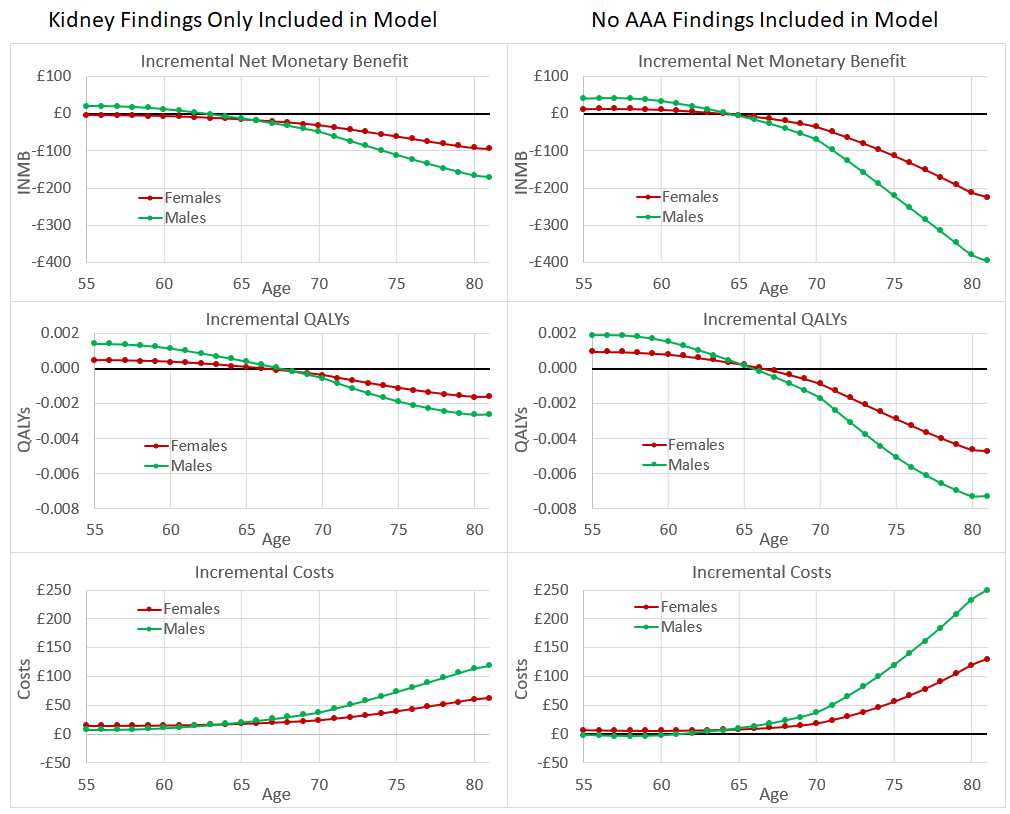

Supplement: Supplementary file 3 — Supplementary Results Tables and Figures [file 41416_2025_3043_MOESM3_ESM.docx]
